# Comparison of the effectiveness of Martin’s equation, Friedewald’s equation, and a Novel equation in low-density lipoprotein cholesterol estimation

**DOI:** 10.1038/s41598-021-92625-x

**Published:** 2021-06-29

**Authors:** Youhyun Song, Hye Sun Lee, Su Jung Baik, Soyoung Jeon, Donghee Han, Su-Yeon Choi, Eun Ju Chun, Hae-Won Han, Sung Hak Park, Jidong Sung, Hae Ok Jung, Ji Won Lee, Hyuk-Jae Chang

**Affiliations:** 1 Department of Family Medicine, Gangnam Severance Hospital, Yonsei University College of Medicine, 211 Eonju‐ro, Gangnam‐gu, Seoul, 06273 Korea; 2 Biostatistics Collaboration Unit, Yonsei University College of Medicine, 211 Eonju-ro, Gangnam-gu, Seoul, 06273 Korea; 3 Healthcare Research Team, Health Promotion Center, Gangnam Severance Hospital, 211 Eunju-ro, Gangnam-gu, Seoul, 06273 Korea; 4 Department of Imaging and Medicine, Cedars Sinai Medical Center, Los Angeles, CA USA; 5 Division of Cardiology, Seoul National University Healthcare System Gangnam Center, Seoul National University College of Medicine, Seoul, Korea; 6 Department of Radiology, Seoul National University Bundang Hospital, Seoul, Korea; 7 Department of Internal Medicine, Gangnam Heartscan Clinic, Seoul, Korea; 8 Department of Radiology, Gangnam Heartscan Clinic, Seoul, Korea; 9 Division of Cardiology, Department of Medicine, Sungkyunkwan University School of Medicine, Heart Stroke & Vascular Institute, Samsung Medical Center, Seoul, Korea; 10 Division of Cardiology, Cardiovascular Center, College of Medicine, Seoul St. Mary’s Hospital, The Catholic University of Korea, Seoul, Korea; 11 Division of Cardiology, Severance Cardiovascular Hospital, Yonsei University College of Medicine, 50-1 Yonsei-ro, Seodaemun-gu, Seoul, 03722 Korea

**Keywords:** Cardiovascular diseases, Dyslipidaemias, Risk factors, Preventive medicine, Lipids

## Abstract

Low-density-lipoprotein cholesterol (LDL-C) is the main target in atherosclerotic cardiovascular disease (ASCVD). We aimed to validate and compare a new LDL-C estimation equation with other well-known equations. 177,111 samples were analysed from two contemporary population-based cohorts comprising asymptomatic Korean adults who underwent medical examinations. Performances of the Friedewald (FLDL), Martin (MLDL), and Sampson (SLDL) equations in estimating direct LDL-C by homogenous assay were assessed by measures of concordance (R^2^, RMSE, and mean absolute difference). Analyses were performed according to various triglyceride (TG) and/or LDL-C strata. Secondary analyses were conducted within dyslipidaemia populations of each database. MLDL was superior or at least similar to other equations regardless of TG/LDL-C, in both the general and dyslipidaemia populations (RMSE = 11.45/9.20 mg/dL; R^2^ = 0.88/0.91; vs FLDL: RMSE = 13.66/10.42 mg/dL; R^2^ = 0.82/0.89; vs SLDL: RMSE = 12.36/9.39 mg/dL; R^2^ = 0.85/0.91, per Gangnam Severance Hospital Check-up/Korea Initiatives on Coronary Artery Calcification data). MLDL had a slight advantage over SLDL with the lowest MADs across the full spectrum of TG levels, whether divided into severe hyper/non-hyper to moderate hypertriglyceridaemia samples or stratified by 100-mg/dL TG intervals, even up to TG values of 500–600 mg/dL. MLDL may be a readily adoptable and cost-effective alternative to direct LDL-C measurement, irrespective of dyslipidaemia status. In populations with relatively high prevalence of mild-to-moderate hypertriglyceridaemia, Martin’s equation may be optimal for LDL-C and ASCVD risk estimation.

## Introduction

Low-density lipoprotein cholesterol (LDL-C) is a primary therapeutic target in the prevention of cardiovascular disease (CVD). In a previous meta-analysis, the relative risk of major vascular events was proportionally reduced even at normal LDL-C levels and each 39-mg/dL reduction in this level reduced the risk of cardiovascular events by about a fifth^1^. Recent American Heart Association/American College of Cardiology (AHA/ACC) and European Society of Cardiology/European Atherosclerosis Society (ESC/EAS) guidelines emphasise the use of aggressive LDL-C targeted therapy for reductions in the level of LDL-C by > 50% if the level is higher than a certain threshold in very high-risk patients^2–4^. Therefore, accurate LDL-C measurement is important in therapy-related decision-making and planning in clinical practice.

Several equations for LDL-C estimation are generally utilised when direct measurement is unavailable or expensive^5^. Traditionally, LDL-C is widely estimated using the Friedewald equation (FLDL). However, this equation is associated with very low-density lipoprotein cholesterol (VLDL-C) overestimation and LDL-C underestimation under conditions of low LDL-C and high triglyceride (TG) levels, given the fixed TG:VLDL-C ratio of 5:1^6,7^. Inaccurate LDL-C estimation may lead to cardiovascular risk misclassification. Therefore, Martin et al. developed an equation using an adjustable factor (strata-specific median VLDL-C:TG ratio) in place of the fixed TG denominator of 5^8^. This equation is more accurate than the Friedewald equation, particularly at low LDL-C levels, and shows a much stronger concordance with directly measured LDL-C using the ultracentrifugation method than the Friedewald equation, according to TG level^9^. Nonetheless, the Martin/Hopkins LDL-C (MLDL) equation (or Martin’s equation) does still tend to overestimate the LDL-C level (or direct LDL-C [dLDL]) at high TG concentrations.

Recently, Sampson et al. derived a novel LDL-C (SLDL) equation using the United States (US) National Institutes of Health database, including 18,715 samples from 8656 patients. The new equation, which they deemed particularly favourable for use in patients with low levels of LDL-C and/or hypertriglyceridemia, yielded a misclassification rate lower than 35% in the categorisation of patients with hypertriglyceridemia into different LDL-C treatment groups^5^.

For the adoption of new methods, the principles of evidence-based medicine require external validation in independent populations on the basis of various race/ethnicities and the use of other laboratory techniques. To the best of our knowledge, no study till date has validated the Sampson equation in Asian populations. Accordingly, this study aimed to validate and compare the performance of the Friedewald, Martin, and Sampson equations in LDL-C estimation with a direct homogeneous assay in the Korean population.

## Methods

### Study population

This study used data from two large population-based databases: the Gangnam Severance Hospital Health Promotion Center Cohort (Gangnam Severance Hospital Check-up [GSHC]) and the Korea Initiatives on Coronary Artery Calcification (KOICA) registry. A total of 79,467 individuals (Korean adults aged ≥ 19 years) underwent in-depth medical examinations in the Gangnam Severance Hospital between 2nd March 2007 and 12th March 2020; this database comprises 144,910 samples. The retrospective, multicentre, observational KOICA registry was designed for the evaluation of the value of coronary artery calcification scores in the prediction of CVD in asymptomatic Korean adults. A total of 48,901 participants were initially enrolled between December 2002 and July 2014; the database comprises 56,446 samples. Details on the GSHC^10^ and KOICA^11^ have been provided elsewhere. The populations of both databases comprise self-referred individuals who underwent general health check-ups at healthcare centres in Seoul, South Korea, and information on their personal medical history and data were obtained by self-reported questionnaires. All participants voluntarily signed an informed consent form before the study, and the institutional review boards (IRB) of each study site approved the study protocols.

**Figure 1:**
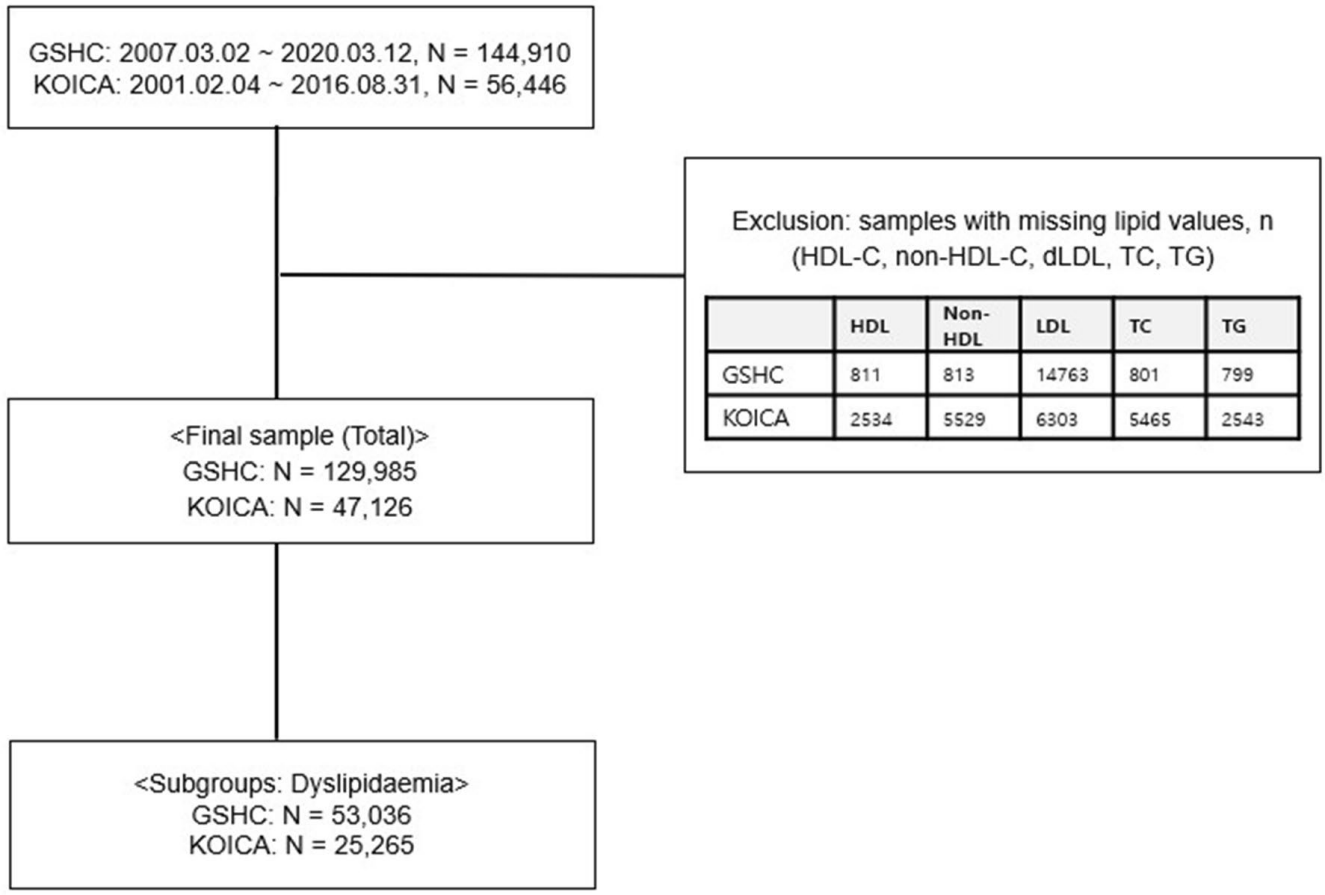
Flowchart of study design. *N* number; *HDL-C* high-density lipoprotein cholesterol; *Non-HDL-C* non-high-density lipoprotein cholesterol; *TC* total cholesterol; *LDL-C* low-density lipoprotein cholesterol; *dLDL* direct LDL-C; *TG* triglyceride; *GSHC* Gangnam Severance Hospital Check-up; *KOICA* Korea Initiatives on Coronary Artery Calcification.

After excluding samples with missing lipid values, a total of 177,111 samples were included—129,985 cases from the GSHC and 47,126 cases from the KOICA. Figure 1 presents a flow chart of the study process. Secondary analyses were performed in the subgroups of participants who met the diagnostic criteria for dyslipidaemia in each database (53,036 cases from the GSHC and 25,265 from the KOICA) for the additional validation and comparison of the three equations in individuals with dyslipidaemia. Additional sensitivity analyses were performed after exclusion of the highest and lowest 0.5 percentiles of each lipid parameter (total cholesterol [TC], high-density lipoprotein cholesterol [HDL-C], TG, non-HDL-C, and LDL-C) (See Supplementary File 2 for full results). This study was conducted in accordance with the Declaration of Helsinki and approved by the Institutional Review Board of Severance Hospital (IRB No: 4-2020-0323).

### LDL-C estimation

The level of LDL-C was estimated using the Friedewald, Martin, and Sampson equations, which are:

$$ {\text{Friedewald}}\;{\text{equation}}:{\text{ LDL}} - {\text{C}} = {\text{TC}} - {\text{HDL}} - {\text{C}} - {\text{TG}}/5 $$

$$ {\text{Martin}}\,{\text{equation: LDL}} - {\text{C}} = {\text{TC}} - {\text{HDL}} - {\text{C}} - {\text{TG}}/{\text{adjustable}}\;{\text{factor}}\;\left( {{\text{based}}\;{\text{on}}\;{\text{the}}\;{\text{non - HDL - C}}\;{\text{and}}\;{\text{TG}}\;{\text{levels}}\;{\text{derived}}\;{\text{from}}\;{\text{a}}\;180 - {\text{cell}}\;2{\text{D}}\;{\text{table}}} \right) $$

$$ {\text{Sampson}}\;{\text{equation}}:{\text{LDL - C}} = {\text{TC}}/0.948 - {\text{HDL - C}}/0.971 - \left( {{\text{TG}}/8.56 + \left[ {{\text{TG}}*{\text{non - HDL - C}}} \right]/2140 - {\text{TG}}^{2} /16100} \right) - 9.44 $$

### Definition of dyslipidaemia

Dyslipidaemia was defined on the basis of the presence of a diagnosis by a physician, the current use of lipid-lowering medications, or National Cholesterol Education Program (NCEP)—Adult Treatment Panel III criteria: (1) hypercholesterolaemia (serum TC ≥ 240 mg/dL), (2) hypertriglyceridemia (serum TG ≥ 150 mg/dL), (3) hyper-LDL-cholesterolaemia (serum LDL-C ≥ 160 mg/dL), or (4) hypo-HDL-cholesterolaemia (serum HDL-C < 40 mg/dL).

### Lipid measurements

Blood samples were collected after an eight-hour fast, at minimum, in both cohorts. In the GSHC, serum LDL-C was measured by a homogenous direct assay using reagents from Sekisui Medical Corporation (Tokyo, Japan) on a Hitachi 7600 automated analyser (Hitachi, Tokyo, Japan) until 17th March 2014 and then by a homogeneous direct assay using reagents from Beckman Coulter Inc. (Brea, CA, USA) on an AU5800 automated analyser (Beckman Coulter Inc.) from 18th March 2014. In the KOICA registry, data were gathered from three locations: Severance Check-up Healthcare Center, Seoul National University Healthcare System Gangnam Center, and Samsung Medical Center. Serum LDL-C levels were measured by homogenous direct assays using reagents from Sekisui, Beckman, or Roche Diagnostics (Mannheim, Germany) on Hitachi 7600, Modular D2400, or Architect Ci8200 (Abbott, Abbott Park, IL) automated analysers. Further details of lipid measurements are shown in Supplementary Table S4 (File 1).

The departments of laboratory medicine at all the sites have been accredited by the Korean Society of Laboratory Medicine and participate in annual inspections administered by the Korean Association of Quality Assurance for Clinical Laboratories as well as Proficiency Testing surveys provided by the College of American Pathologists.

### Statistical analysis

Continuous variables are reported as mean (standard deviation), median (interquartile range [IQR]), and range. Categorical variables are shown as number (%).

The relationships between the three equations and dLDL were visually assessed with scatter plots, and the formulae were derived by linear regression. Concordance was evaluated with R^2^ and root mean square error (RMSE). The difference between the dLDL and LDL-C estimations obtained according to high/low TG or LDL-C levels, stratified by TG or LDL-C levels across the ranges of each value were used to draw residual error plots, following which mean absolute difference (MAD) values were evaluated.

All analyses were performed using data on both the general and dyslipidaemia populations with SAS 9.2 (SAS Institute, Cary, NC). Two-sided P values < 0.05 were considered statistically significant.

## Results

The distribution of lipid values of the patients (from both the general and dyslipidaemia populations) in the GSHC and KOICA databases are presented in Table 1. A total of 129,985 samples were included in the GSHC database (53.53% male; age 48.58 [11.46] years) and 47,126 in the KOICA database (76.04% male; age 54.05 [8.88] years); the prevalence rates of dyslipidaemia were 53,036 (40.80%) and 25,265 (53.61%), respectively. In the GSHC (ranges, TG: 8–3271 mg/dL; LDL-C: 10–386 mg/dL), 1.32% of the samples had TG levels ≥ 400 mg/dL, 22.3% had LDL-C values < 100 mg/dL, and 0.52% had LDL-C levels ≥ 220 mg/dL; in the KOICA (ranges, TG: 16–2309 mg/dL; LDL-C: 11–356 mg/dL) the corresponding percentages were 1.42%, 21.4%, and 0.38%, respectively.

**Table 1:** Distribution of lipid parameters of the study populations.

Database	Total cohort			Dyslipidaemia		
	Range	Mean (SD)	Median (25th–75th percentile)	Range	Mean (SD)	Median (25th–75th percentile)
**GSHC**						
Cases, n (%)	129,985 (100.00)			53,036 (40.80)		
Male, n (%)	69,575 (53.53)			35,978 (67.84)		
Age, year	9–201	54.42 (13.14)	53 (45–62)	9–201	49.1 (13.22)	47 (39–56)
HDL-C, mg/dL	29–622	143.89 (36.41)	142 (118–167)	38–622	167.32 (36.7)	170 (143–191)
Non-HDL-C, mg/dL	10–386	124.82 (32.4)	123 (102–146)	15–386	141.52 (35.67)	143 (116–166)
Direct LDL-C, mg/dL	77–696	198.31 (37.03)	196 (173–221)	77–696	216.42 (42.01)	219 (187–246)
TC, mg/dL	8–3271	127.4 (85.5)	105 (75–153)	23–3271	184.72 (105.97)	167 (122–217)
TG, mg/dL	9–201	54.42 (13.14)	53 (45–62)	9–201	49.1 (13.22)	47 (39–56)
Cases by TG range, n (%)						
0–400 mg/dL	128,271 (98.68)			51,322 (96.77)		
≥ 400 mg/dL	1714 (1.32)			1714 (3.23)		
≥ 600 mg/dL	422 (0.32)			422 (0.80)		
Cases by LDL-C range, n (%)						
< 40 mg/dL	111 (0.09)			39 (0.07)		
40– 100 mg/dL	28,842 (22.19)			6880 (12.97)		
≥ 100 mg/dL	101,032 (77.73)			46,117 (86.95)		
≥ 220 mg/dL	678 (0.52)			678 (1.29)		
Estimated LDL-C values						
FLDL, mg/dL	− 214.2 to 370.2	118.41 (33.37)	116.8 (95.6–139.6)	− 214.2 to 370.2	130.38 (40.07)	131.4 (102–159.6)
MLDL, mg/dL	− 89.31 to 366.98	120.38 (32.13)	118.49 (97.92–140.86)	− 89.31 to 366.98	136.3 (35.28)	137.38 (111.03–161)
SLDL, mg/dL	0.42–374.33	121.13 (32.99)	119.34 (98.15–142.11)	0.42–374.33	134.86 (37.86)	135.5 (107.31–162.29)
**KOICA**						
Cases, n (%)	47,126 (100.00)			25,265 (53.61)		
Male, n (%)	35,835 (76.04)			20,281 (80.27)		
Age, year	16–97	54.05 (8.88)	53 (48–59)	17–97	54.03 (8.53)	53 (48–59)
HDL-C, mg/dL	13–162	52.33 (13.11)	50 (43–60)	13–162	48.35 (12.68)	46 (39–55)
Non-HDL-C, mg/dL	31–437	144.8 (34.81)	143 (121–167)	31–437	157.55 (36.83)	158 (132–183)
Direct LDL-C, mg/dL	11–356	124.6 (31.34)	123 (103–145)	11–356	132.06 (35.1)	131 (106–159)
TC, mg/dL	73–450	197.13 (35.04)	196 (173–219)	73–450	205.9 (39.42)	206 (178–234)
TG, mg/dL	16–2309	133.4 (85.17)	113 (79–163)	20–2309	172.14 (97.75)	158 (109–208)
Cases by TG range, n (%)						
0–400 mg/dL	46,455 (98.58)			24,594 (97.34)		
≥ 400 mg/dL	671 (1.42)			671 (2.66)		
≥ 600 mg/dL	121 (0.26)			121 (0.48)		
Cases by LDL-C range, n (%)						
< 40 mg/dL	45 (0.1)			28 (0.11)		
40–100 mg/dL	10,061 (21.35)			4768 (18.87)		
≥ 100 mg/dL	37,020 (78.56)			20,469 (81.02)		
≥ 220 mg/dL	178 (0.38)			178 (0.70)		
Estimated LDL-C Values						
FLDL, mg/dL	− 122.6 to 370.4	118.12 (32.6)	117 (96–139)	− 122.6 to 370.4	123.12 (37.73)	121.2 (95.8–150.6)
MLDL, mg/dL	− 14.06 to 368	120.62 (31)	119.35 (99.35–140.24)	− 14.06 to 368	128.43 (34.25)	127.23 (103.4–153)
SLDL, mg/dL	3.86–373.66	121.04 (32.02)	119.84 (98.97–141.52)	3.86–373.66	127.24 (36.16)	125.23 (100.74–153.59)

Values are presented as mean (standard deviation), median (interquartile range), or as number (%).

*SD* standard deviation; n, number; *HDL-C* high-density lipoprotein cholesterol; *Non-HDL-C* non-high-density lipoprotein cholesterol; *TC* total cholesterol; *LDL-C* low-density lipoprotein cholesterol; *dLDL* direct LDL-C; *TG* triglyceride; *GSHC* Gangnam Severance Hospital Check-up; *KOICA* Korea Initiatives on Coronary Artery Calcification; *FLDL* LDL-C estimated by Friedewald’s equation; *MLDL* LDL-C estimated by Martin/Hopkins equation; *SLDL* LDL-C estimated by Sampson’s equation.

**Figure 2:**
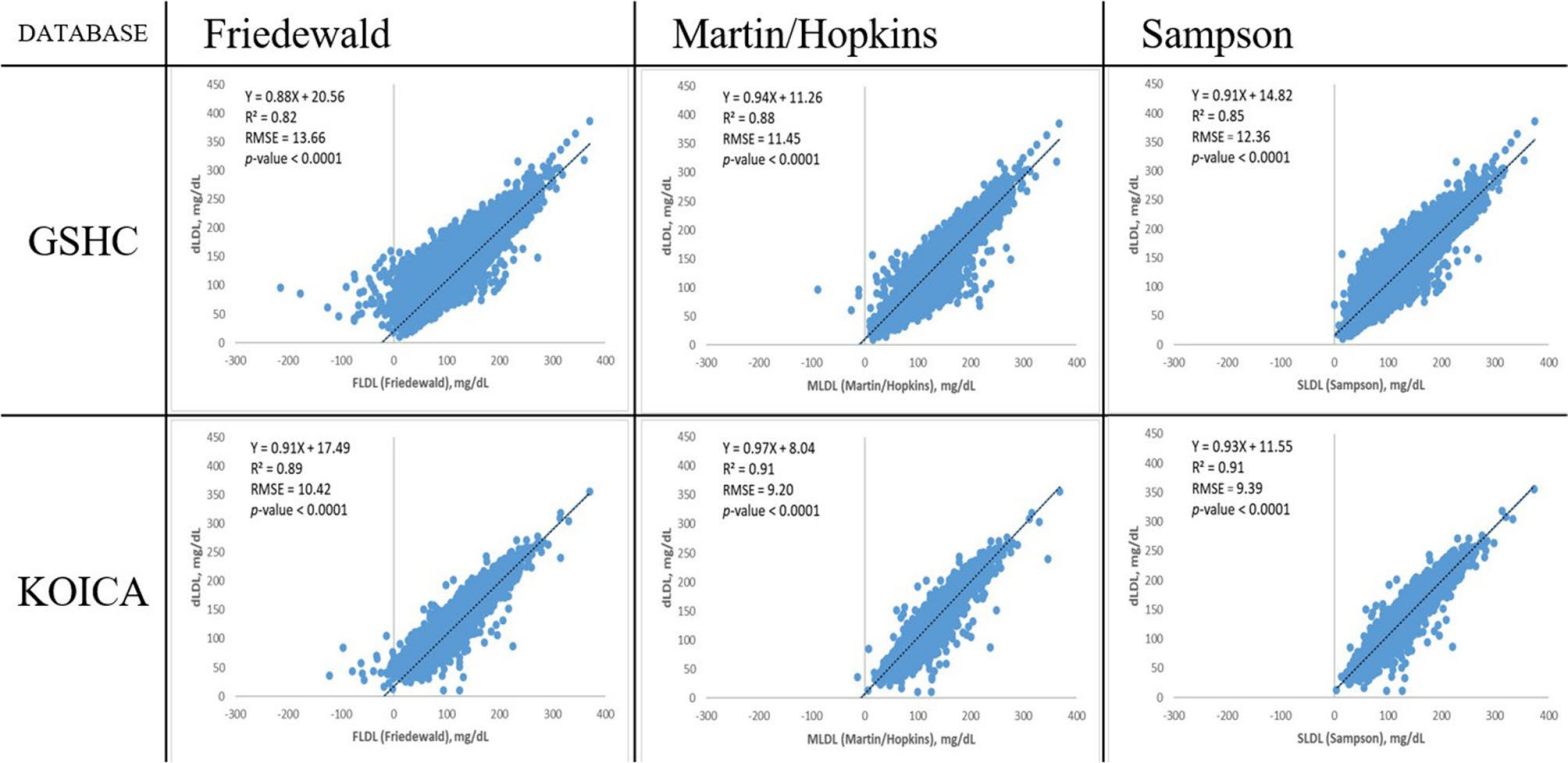
Scatter plots showing the correlation of direct LDL-C values with estimated LDL-C values using the Friedewald, Martin/Hopkins, and Sampson equations. *RMSE* root mean square error; *GSHC* Gangnam Severance Hospital Check-up; *KOICA* Korea Initiatives on Coronary Artery Calcification; *LDL-C* low-density lipoprotein cholesterol; *dLDL* direct LDL-C; *TG* triglyceride.

Figure 2 shows the overall good correlations between the estimated LDL-C values (FLDL, MLDL, and SLDL) and dLDL levels. However, MLDL showed a slightly better fit with dLDL than FLDL or SLDL in both the study groups (slope, 0.94; RMSE = 11.45 mg/dL; R^2^ = 0.88; vs Friedewald equation: slope, 0.88; RMSE = 13.66 mg/dL; R^2^ = 0.82; vs Sampson equation: slope, 0.91; RMSE = 12.36 mg/dL; R^2^ = 0.85 in the GSHC) (slope, 0.97; RMSE = 9.20 mg/dL; R^2^ = 0.91; vs Friedewald equation: slope, 0.91; RMSE = 10.42 mg/dL; R^2^ = 0.89; vs Sampson equation: slope, 0.93; RMSE = 9.39 mg/dL; R^2^ = 0.91 in the KOICA).

Similar results were shown within the dyslipidaemia populations (Supplementary Fig. S1); again, MLDL showed a better fit with dLDL than FLDL, and fit better or similarly to SLDL in both the study groups (slope, 0.93; RMSE = 14.18 mg/dL; R^2^ = 0.84; vs Friedewald equation: slope, 0.80; RMSE = 15.96 mg/dL; R^2^ = 0.80; vs Sampson equation: slope, 0.86; RMSE = 14.84 mg/dL; R^2^ = 0.83 in the GSHC) (slope, 0.98; RMSE = 10.21 mg/dL; R^2^ = 0.92; vs Friedewald equation: slope, 0.88; RMSE = 11.17 mg/dL; R^2^ = 0.90; vs Sampson equation: slope, 0.93; RMSE = 10.15 mg/dL; R^2^ = 0.92 in the KOICA).

**Figure 3:**
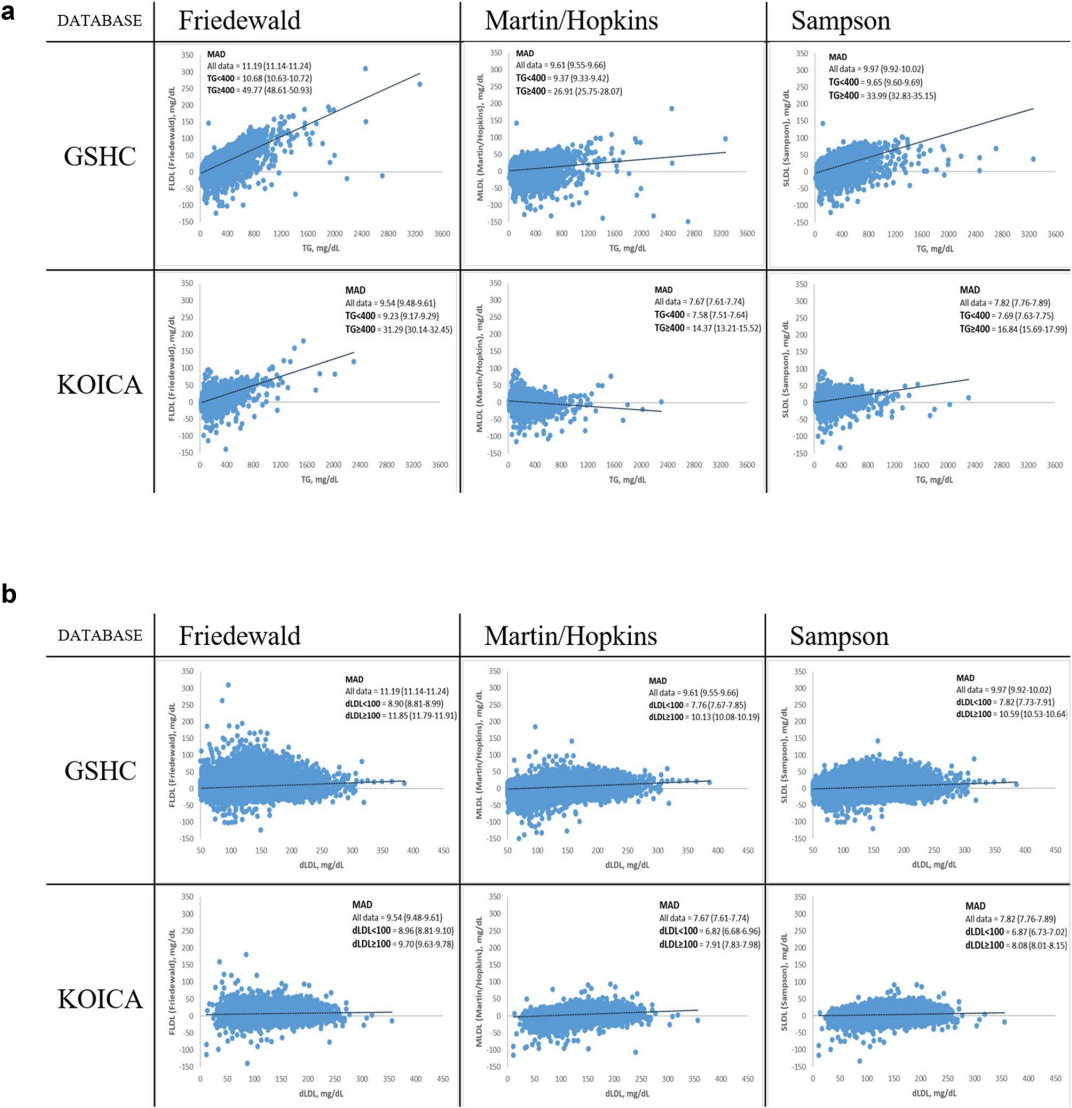
Residual error plots for LDL-C by different equations. (**a**) Severe hyperTG/ TG < 400 mg/dL; (**b**) High/Low LDL-C. *MAD* mean absolute difference; *RMSE* root mean square error; *LDL-C* low-density lipoprotein cholesterol; *dLDL* direct LDL-C; *TG* triglyceride; *GSHC* Gangnam Severance Hospital Check-up; *KOICA* Korea Initiatives on Coronary Artery Calcification.

**Figure 4:**
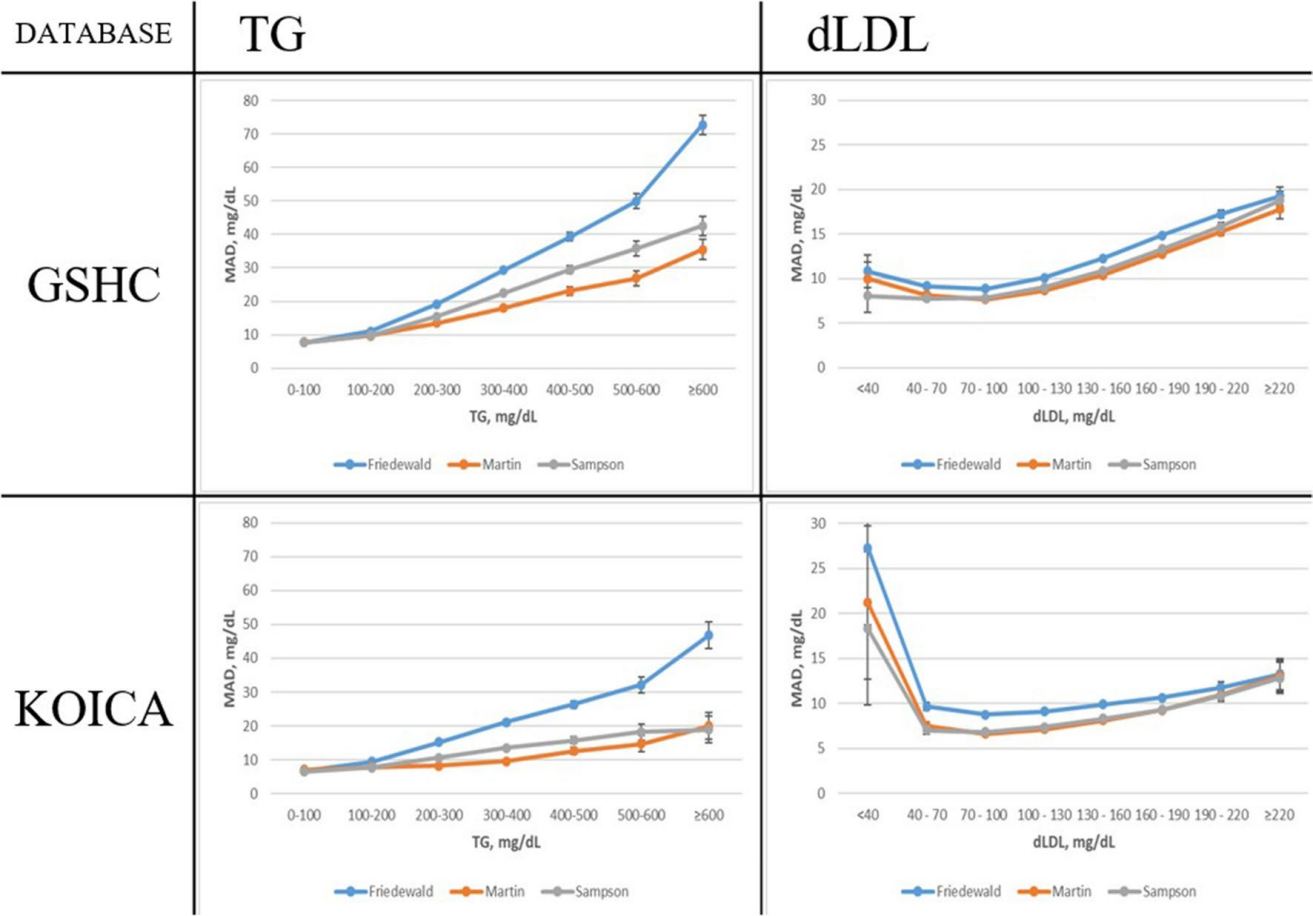
Comparison of the mean absolute difference scores between direct LDL-C and different estimated LDL-C values for various TG and LDL-C levels. *TG* triglyceride; *LDL-C* low-density lipoprotein cholesterol; *GSHC* Gangnam Severance Hospital Check-up; *KOICA* Korea Initiatives on Coronary Artery Calcification.

We compared the three equations with a single integrated index of accuracy by the calculation of the MAD from the dLDL value first divided into samples with high and lower TG levels (severe hypertriglyceridaemia: TG ≥ 400 mg/dL) and LDL-C (low LDL-C: LDL < 100 mg/dL) (Fig. 3), then across the spectrum of TG levels and LDL-C values (Fig. 4). All the results were again validated in the dyslipidaemia populations within both the study groups (Supplementary Figs. S2 and S3). Confidence intervals for calculated MADs are available in Supplementary Tables S2 and S3.

The MAD values for each database in entirety indicated the overall superiority of MLDL (MAD = 9.61 mg/dL; vs Friedewald equation: MAD = 11.19 mg/dL; vs Sampson equation: MAD = 9.97 mg/dL in the GSHC) (MAD = 7.67 mg/dL; vs Friedewald equation: MAD = 9.54 mg/dL; vs Sampson equation: MAD = 7.82 mg/dL in the KOICA), closely followed by SLDL; FLDL showed the poorest results. Similar findings were observed in the dyslipidaemia populations in both the databases (MAD = 11.97 mg/dL; vs Friedewald equation: MAD = 15.58 mg/dL; vs Sampson equation: MAD = 13.06 mg/dL in the GSHC) (MAD = 8.15 mg/dL; vs Friedewald equation: MAD = 11.55 mg/dL; vs Sampson equation: MAD = 8.85 mg/dL in the KOICA).

MLDL was generally associated with the lowest MADs across the full spectrum of TG levels, whether divided into severe hyper/non-hyper to moderate hypertriglyceridaemia samples or stratified by 100-mg/dL TG intervals (Figs. 3A and 4) (MADs for MLDL vs FLDL vs SLDL: TG < 400 mg/dL, 9.37 mg/dL vs 10.68 mg/dL vs 9.65 mg/dL; TG ≥ 400 mg/dL, 26.91 mg/dL vs 49.77 mg/dL vs 33.99 mg/dL in the GSHC, respectively) (MADs for MLDL vs FLDL vs SLDL: TG < 400 mg/dL, 7.58 mg/dL vs 9.23 mg/dL vs 7.69 mg/dL; TG ≥ 400 mg/dL, 14.37 mg/dL vs 31.29 mg/dL vs 16.84 mg/dL in the KOICA, respectively), followed closely by SLDL; FLDL showed the weakest concordance.

When stratified by LDL-C levels across the spectrum, MLDL and SLDL generally showed closely matched superior results, while FLDL showed much lower performance levels (Figs. 3B and 4) (MADs for MLDL vs FLDL vs SLDL: LDL-C < 100 mg/dL, 7.76 mg/dL vs 8.90 mg/dL vs 7.82 mg/dL; LDL-C ≥ 100 mg/dL, 10.13 mg/dL vs 11.85 mg/dL vs 10.59 mg/dL in the GSHC, respectively) (MADs for MLDL vs FLDL vs SLDL: LDL-C < 100 mg/dL, 6.82 mg/dL vs 8.96 mg/dL vs 6.87 mg/dL; LDL-C ≥ 100 mg/dL, 7.91 mg/dL vs 9.70 mg/dL vs 8.08 mg/dL in the KOICA, respectively). Similar results were observed in the dyslipidaemia populations for both the TG and LDL-C strata.

Sensitivity analysis of all computed results was performed after excluding lipid value outliers, and the results in entirety is presented in Supplementary File 2. The overall results were similar to the original analysis, and trends were shown in more clarity.

## Discussion

This study aimed to conduct comparative analyses of the performance of a novel equation and other widely-used equations in LDL-C estimation (Sampson’s, Friedewald’s, and Martin’s equations) with a direct homogeneous assay using two large contemporary real-world cohorts of East Asians.

Overall, we concluded that Martin’s equation accounts for a slightly more superior estimation of LDL-C in Korean adults, both in the general population as well as in people with dyslipidaemia. The new Sampson’s equation closely matches Martin’s equation in performance, while Friedewald’s equation showed the most discordant results. The Martin equation was advantageous throughout all LDL-C and TG strata, even at severe hypertriglyceridaemic levels.

LDL-C level optimisation is among the main targets in the prevention of ASCVD, and substantial progress has been made toward LDL-C quantification. Martin’s equation was considerably superior to Friedewald’s equation, especially under conditions of low LDL-C or elevated TG levels^9^. The 2018 AHA/ACC/Multi-society Cholesterol Guideline provided a Class IIa recommendation for the use of Martin’s equation in patients with LDL-C levels < 70 mg/dL^2^. Additionally, Martin’s equation has been further validated in LDL-C estimation by numerous studies, when LDL-C levels < 70 mg/dL and TG levels are > 150 mg/dL^6,8,12^. However, both the Friedewald and Martin equations were developed and validated for patients with serum TG levels < 400 mg/dL; MLDL also remains imperfect, particularly in cases with severe hypertriglyceridemia^13^. Sampson et al. recently developed a new method for the calculation of LDL-C using β-quantification LDL-C values and multiple least squares regressions analysis. Their equation was reported to have a particularly good performance level in patients with hypertriglyceridemia (TG levels up to 800 mg/dL) and/or low LDL-C levels, and to show similar or slightly higher accuracy values than the other equations in those with normal lipid levels^5^.

Consistent with previous studies, our analyses showed that the Martin and Sampson equations are generally more accurate than Friedewald’s equation.

Interestingly, in our study, Martin’s equation had a slight advantage over Sampson’s equation spanning the whole range of TG levels, and the advantage grew progressively stronger with increasing TG, even at severe hypertriglyceridaemic levels up to 500 ~ 600 mg/dL. Sampson et al. also observed similar accuracy values between the equations but at TG levels lower than 400 mg/dL. On comparing the performance of the Martin and Sampson equations at different LDL-C levels, the results showed similar levels of superiority over Friedewald’s equation. Sampson et al. also showed similar accuracy values between the equations at low LDL-C levels; however, in their study, SLDL began gaining an advantage over MLDL at an LDL-C level of approximately 100 mg/dL and progressively increased at higher LDL-C levels, as observed by the MAD values. This discrepancy warrants further validation since the Sampson equation may substantially underestimate LDL-C at low levels, as commented by Martin et al.^14^.

Several possibilities, including multifactorial differences across ethnicities, such as those pertaining to genetics or associated lifestyles may have contributed to our finding on the superiority of MLDL according to TG strata even at high TG levels, potentially explaining other minor discrepancies between the results of the study conducted by Sampson et al. and our study. The likeliest variable is the patient sample that was used in the derivation of the new equation, which differed significantly from that employed in this study. Sampson et al. used 18,715 lipid samples from 8656 patients, as collected at the US National Institutes of Health for the derivation and external validation of their equation in multiple US datasets. Additionally, their derivation database included higher TG and non-HDL-C levels than those in the general US population, including extremely high TG levels of up to 3162 mg/dL, with 14% of the samples showing TG levels higher than 400 mg/dL. Our two Korean-based databases comprised significantly lower percentages of TG levels ≥ 400 mg/dL (1.32% and 1.42%).

However, it is important to note that most widely accepted treatment guidelines or risk calculation tools (such as the Pooled Cohort Equation, criteria for metabolic syndrome, etc.) have been developed on the basis of Western (mainly European and North American) populations, as has Sampson’s equation. Several studies that validated the application of such recommendations in non-Western populations showed discrepant results^15–18^. Major societies in medicine have begun voicing the need for exercising caution in the extension of the same guidelines to other populations without supporting research^19,20^; most recently, for example, the 2018/2019 ACC/AHA guidelines stated that race and ethnicity influence the risk of CVD and choice of treatment (Class IIa)^3^. Our study is significant in that, to the best of our knowledge, it is the first to validate Sampson’s equation in an East Asian population.

Growing evidence suggests that high TG levels (by reflecting the number of triglyceride-rich lipoproteins [TRLs] and their remnants) are independent risk factors for CVD, at low HDL-C levels or otherwise^16,21–26^. TRLs are hydrolysed into remnant-like lipoprotein particles, which are considered as atherogenic as LDL-C and as being associated with atherogenesis^27^. New epidemiological and genetic insights as well as in-vitro/animal studies suggest that TRLs are causal risk factors for low-grade inflammation, atherosclerosis, ASCVD, and all-cause mortality, as opposed to LDL-C, causing atherosclerosis without a significant inflammatory component^21,22,25,27^. Furthermore, numerous studies have indicated that a high TG level in itself is associated with insulin resistance, obesity, diabetes, and ultimately metabolic syndrome, and when concurrent with low HDL-C, which is more commonly observed in East Asians, demonstrates a high degree of atherogenicity^28^. Such associations generally appear consistently among diverse populations, but the relative strength of the correlations differ by race or ethnicity^29^.

East Asians are known to have lower LDL-C levels and higher TG levels than North Americans and Europeans^19,20,30–32^. Koreans show a strong tendency towards hypertriglyceridaemia development, weak LDL-C distribution, as well as significantly low HDL-C levels. Over the last two decades, Koreans’ TC and LDL-C levels have progressively increased (albeit still relatively lower than those among their Western counterparts), and the trend of high TG and low HDL-C levels have become significantly more pronounced^15,33^. The reasons for this may be multi-faceted: (1) Korean dietary patterns are characterised by significantly higher carbohydrate levels and lower fat proportions than those in Western countries (as per the 2017 statistics provided by the Korean Centers for Disease Control and Prevention, the average Korean diet comprises 62.4% carbohydrates and 22.5% fat; the corresponding numbers in the US were 47.3% and 34.8%, respectively)^34^. The consumption of carbohydrates in the place of fats leads to decreases in the levels of LDL-C and HDL-C and increases in the level of TG^35^, especially in terms of carbohydrate-rich foods that comprise a major proportion of a Korean’s diet; (2) Population-specific genetic factors may have a significant effect; large-scale genetic association studies over the past few years have been identifying new, independent, and/or population-specific lipid loci as well as evaluating potential gene-environment interactions with the goal of creating more informed genetic risk models according to population type^36–38^; and (3) Differences related to race/ethnicity, including lifestyle factors, not only in terms of diet but also including factors such as a relatively sedentary culture^15^.

According to 2019 ESC/EAS guidelines, the level of plasma TG, in addition to LDL-C, should be assessed in individuals who may have a higher risk of ASCVD; East Asians, who have higher TG and lower LDL-C levels than Caucasians, may have underestimated ASCVD risk, leading to the erroneous conclusion of non-eligibility for prophylactic statin treatment^4,13,22^. Moreover, recent studies performed in Asian populations showed that serum TG was a better predictor of CVD than LDL-C, suggesting the possibility of the stronger importance of hypertriglyceridaemia over LDL-C in Asians than in Westerners^16–18,24,29^.

Thus, we conclude that Martin’s equation, which fits in a superior manner with dLDL across the wide spectrum of TG, may be the best equation for LDL-C level estimation and accurate ASCVD risk calculation in Korean adults both in the general population and those with dyslipidaemia. As there is no clear explanation to definitively verify the cause-effect of our findings, further validation using large databases of multiple race/ethnicities are warranted, preferably in the form of longitudinal prospective observational studies or randomised controlled trials. Analyses with β-quantification LDL-C in samples with very high TG and/or very low LDL-C levels would seem essential.

### Limitations and strengths

Our study has some limitations. First, we used direct homogenous assays instead of the β-quantification method, which is considered the gold standard for LDL-C measurement^2^. The direct homogeneous methods have been reported to lack specificity for LDL-C, in some cases measuring up to 20% of VLDL^39–41^. However, our automated methods are well-suited to routine clinical application and have an assay precision generally within the level stated in NCEP guidelines^42^. Additionally, compared to ultracentrifugation methods, which require specialised laboratories, direct homogenous assays are readily available for automatic analysis and are, therefore, widely implemented in Korea. The Committee of Clinical Practice Guidelines of the Korean Society of Lipid and Atherosclerosis generally recommends the use of direct assays at a TG level ≥ 400 mg/dL, except in cases requiring critical accuracy^43^. In addition, 2019 EAS/ESC Guidelines acknowledge that both homogenous enzymatic methods and ultracentrifugation for direct LDL-C measurement are useful in such settings^4^. Considering the real-world medical environment in Korea, our analyses using homogenous assays bear practical merit. Second, these findings are specific to the Korean population. Differences in race and the related dietary patterns may have affected the results; further validation is needed to generalise these results to other races and ethnicities^44^. Lastly, due to the limitation of medical history acquired through questionnaires, accurate information regarding use of lipid-lowering medications was limited. Further studies investigating potential differences between medicated populations are warranted.

However, our study has significant strengths: (1) in our analysis, we used two large contemporary real-world databases that adequately reflect the lipid distributions and characteristics of the average individual one would most commonly encounter in a clinical setting; (2) sensitivity analyses and validation were also performed in participants with dyslipidaemia, a population eligible for statin therapy and in which accurate LDL-C estimations are more significant, as well as dual analyses both including and excluding lipid value outliers; and (3) to the best of our knowledge, our study is the first to validate Sampson’s equation and compare its effectiveness with that of direct LDL-C measurement in a large real-world cohort, as well as the first of its kind conducted in an East Asian population.

## Conclusion

In conclusion, we validated and compared Sampson’s equation for LDL-C with the Martin and Friedewald equations in an East Asian population. Martin’s equation could be a cost-effective alternative to direct LDL-C measurement, which may be readily adoptable in clinical laboratories, irrespective of the presence of dyslipidaemia.

In Korean adults, among whom the prevalence of mild-to-moderate hypertriglyceridaemia is relatively high, Martin’s equation may be the best method for the estimation of LDL-C. Further validation in other populations with β-quantification LDL-C are warranted.

## Supplementary Information

Supplementary Information 1.

Supplementary Information 2.

## Data Availability

The data underlying this article is available upon reasonable request to the corresponding authors.
