# Supplementary material for: Comparison of the effectiveness of Martin’s equation, Friedewald’s equation, and a Novel equation in low-density lipoprotein cholesterol estimation
Source: Sci Rep. 2021 Jun 29;11:13545. doi: 10.1038/s41598-021-92625-x (PMC8241859; doi:10.1038/s41598-021-92625-x)
Supplement: Supplementary file 1 — Supplementary Information 1. [file 41598_2021_92625_MOESM1_ESM.docx]

**Comparison of the Effectiveness of Martin’s Equation, Friedewald’s Equation, and a Novel Equation in Low-Density Lipoprotein Cholesterol Estimation**

*Running title: Novel and known formulas for LDL-C estimation*

Youhyun Song, M.D.^†^, Hye Sun Lee, Ph.D.^†^, Su Jung Baik, M.D., Soyoung Jeon, M.S., Donghee Han, M.D., Su-Yeon Choi, M.D., Ph.D., Eun Ju Chun, M.D., Ph.D., Hae-Won Han, M.D., Ph.D., Sung Hak Park, M.D., Ph.D., Jidong Sung, M.D., Hae Ok Jung, M.D., Ph.D.,

Ji Won Lee, M.D., Ph.D.^*^, and Hyuk-Jae Chang, M.D., Ph.D.^*^

† These authors are co‐first authors who equally contributed to this work.

* Co-corresponding authors

**Supplemental Material: File 1**

**Supplementary Figure 1.** Scatter plots showing the correlation of direct LDL-C values with estimated LDL-C values using the Friedewald, Martin/Hopkins, and Sampson equations in dyslipidaemia subjects.

**Supplementary Figure 2.** Residual error plots for LDL-C by different equations in dyslipidaemia subjects.

**A)** Severe hyperTG/ TG < 400 mg/dL; **B)** High/Low LDL-C

**Supplementary Figure 3.** Comparison of the mean absolute difference scores between direct LDL-C and different estimated LDL-C values for various TG and LDL-C levels in dyslipidaemia subjects.

**Supplementary Table 1.** MAD scores between direct LDL-C and estimated LDL-Cs in severe hyperTG ( ≥ 400 mg/dL) samples.

**Supplementary Table 2.** MAD scores between direct LDL-C and different estimated LDL-C values for various TG and LDL-C levels. (*for Figure 3 in the main manuscript*)

**Supplementary Table 3.** MAD scores between direct LDL-C and different estimated LDL-C values for various TG and LDL-C levels in dyslipidaemia subjects. (*for Supplementary Figure 2)*

**Supplementary Table 4.** Lipid measurements.

**Supplementary References.**

**Supplementary Figure 1.** Scatter plots showing the correlation of direct LDL-C values with estimated LDL-C values using the Friedewald, Martin/Hopkins, and Sampson equations in dyslipidaemia subjects.

| 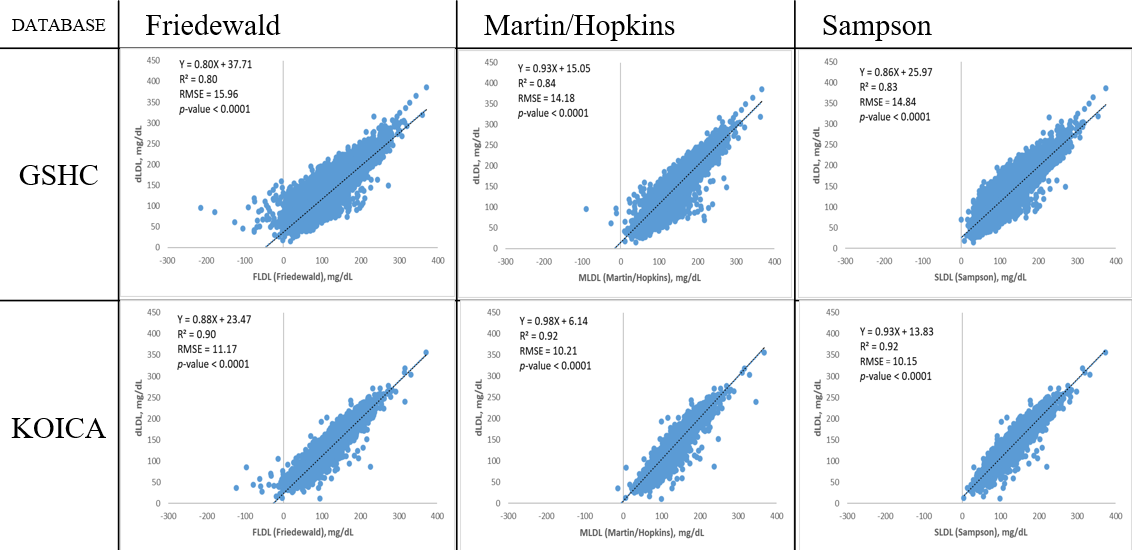 **Supplementary Figure 2.** Residual error plots for LDL-C by different equations in dyslipidaemia subjects  **A)** Severe hyperTG/ TG < 400 mg/dL 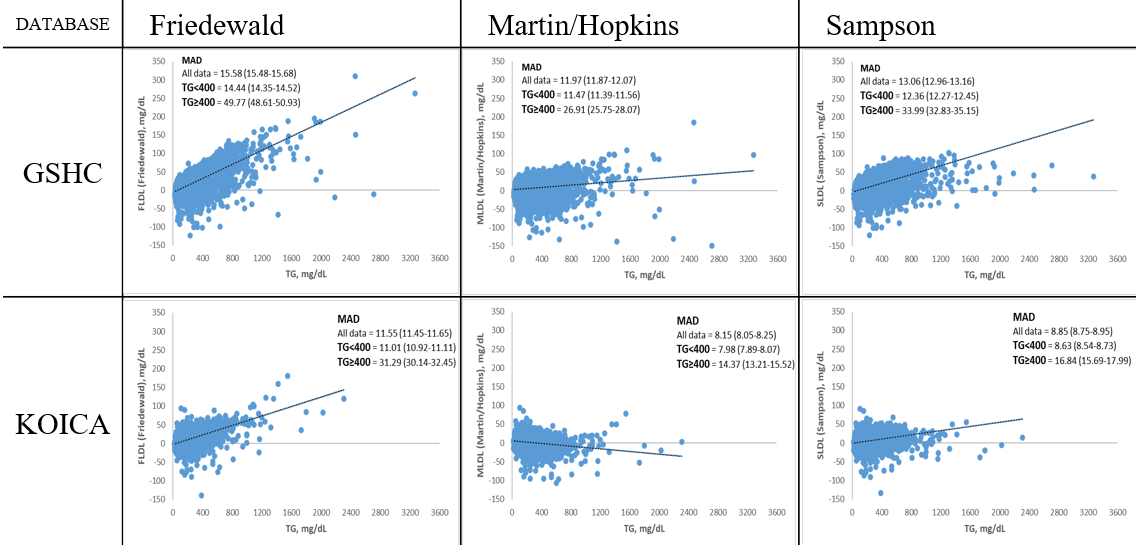 **B)** High/Low LDL-C 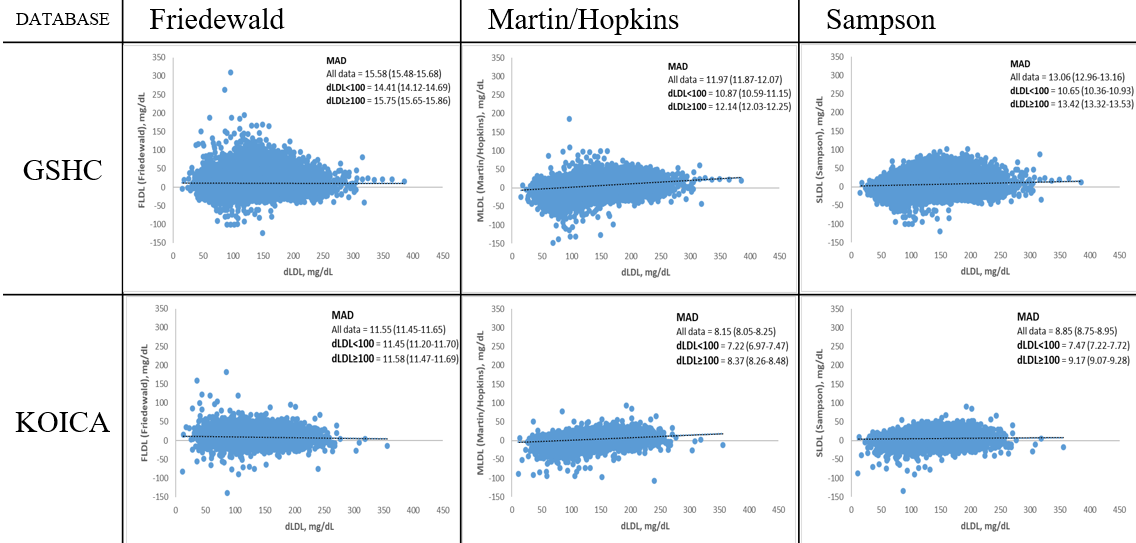 **Supplementary Figure 3.** Comparison of the mean absolute difference scores between direct LDL-C and different estimated LDL-C values for various TG and LDL-C levels in dyslipidaemia subjects 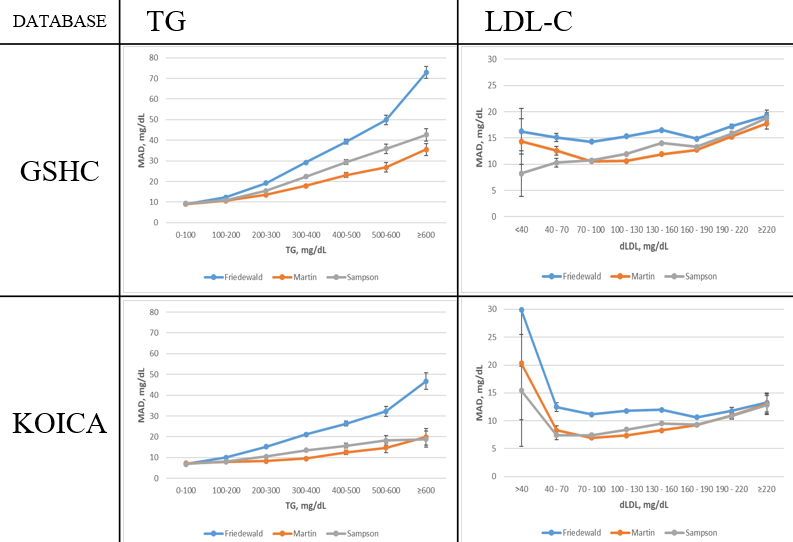 |
| --- |

**Supplementary Table 1.** MAD scores between direct LDL-C and estimated LDL-Cs in severe hyperTG ( ≥ 400 mg/dL) samples

| **Mean absolute difference, mg/dL** | | | | | | |
| --- | --- | --- | --- | --- | --- | --- |
| *Database* | GSHC | | | KOICA | | |
| *Equation* | Friedewald | Martin | Sampson | Friedewald | Martin | Sampson |
| *LDL-C strata, mg/dL* | | | | | | |
| < 40 | 35.58  (22.01-49.15) | 27.99  (14.42-41.56) | 8.91  (-4.66-22.48) | 61.08  (37.42-84.73) | 37.17  (13.52-60.82) | 21.69  (-1.96-45.34) |
| 40-70 | 36.63  (32.57-40.68) | 26.62  (22.56-30.67) | 13.48  (9.42-17.53) | 36.69  (31.94-41.44) | 23.02  (18.28-27.77) | 13.57  (8.82-18.32) |
| 70-100 | 32.82  (30.15-35.50) | 25.86  (23.18-28.53) | 18.05  (15.37-20.72) | 29.98  (27.89-32.08) | 15.70  (13.60-17.79) | 13.22  (11.12-15.31) |
| 100-130 | 49.19  (46.99-51.40) | 24.13  (21.92-26.33) | 31.18  (28.97-33.38) | 29.28  (27.61-30.95) | 12.22  (10.55-13.89) | 15.73  (14.06-17.40) |
| 130-160 | 59.68  (57.71-61.65) | 27.07  (25.10-29.04) | 42.98  (41.01-44.95) | 31.72  (29.51-33.92) | 10.07  (7.87-12.28) | 21.33  (19.13-23.53) |
| 160-190 | 59.49  (57.02-61.97) | 30.49  (28.02-32.97) | 48.19  (45.71-50.67) | 28.53  (24.60-32.46) | 11.74  (7.81-15.67) | 23.17  (19.24-27.10) |
| 190-220 | 59.15  (54.92-63.39) | 33.69  (29.45-37.92) | 52.95  (48.71-57.18) | 35.55  (30.34-40.76) | 12.51  (7.30-17.72) | 33.86  (28.65-39.08) |
| ≥ 220 | 59.07  (51.05-67.09) | 36.44  (28.42-44.46) | 59.99  (51.97-68.01) | 75.80 (-)* | 106.55 (-)* | 40.12 (-)* |

* Confidence interval incomputable due to only 1 sample.

**Supplementary Table 2.** MAD scores between direct LDL-C and different estimated LDL-C values for various TG and LDL-C levels (*for Figure 3 in the main manuscript*)

| MAD, mg/dL (95% CI) | GSHC | | | KOICA | | |
| --- | --- | --- | --- | --- | --- | --- |
|  | Friedewald | Martin | Sampson | Friedewald | Martin | Sampson |
| TG, mg/dL |  |  |  |  |  |  |
| 0 - 100 | 7.65(7.61-7.70) | 7.76(7.72-7.81) | 7.67(7.63-7.72) | 6.63(6.55-6.70) | 7.01(6.93-7.08) | 6.51(6.43-6.59) |
| 100 - 200 | 11.04(10.98-11.11) | 9.74(9.67-9.80) | 9.80(9.74-9.86) | 9.45(9.36-9.54) | 7.82(7.72-7.91) | 7.73(7.64-7.82) |
| 200 - 300 | 19.20(19.00-19.40) | 13.49(13.29-13.70) | 15.42(15.22-15.62) | 15.18(14.96-15.40) | 8.27(8.05-8.49) | 10.55(10.33-10.77) |
| 300 - 400 | 29.23(28.69-29.77) | 17.90(17.36-18.44) | 22.41(21.87-22.94) | 21.17(20.61-21.73) | 9.54(8.98-10.10) | 13.49(12.93-14.05) |
| 400 - 500 | 39.31(38.09-40.52) | 23.05(21.83-24.26) | 29.40(28.19-30.62) | 26.38(25.27-27.49) | 12.58(11.47-13.69) | 15.73(14.63-16.84) |
| 500 - 600 | 49.85(47.63-52.08) | 26.85(24.62-29.08) | 35.82(33.59-38.05) | 32.19(29.82-34.57) | 14.70(12.32-17.08) | 18.27(15.89-20.65) |
| ≥ 600 | 72.82(69.92-75.73) | 35.50(32.60-38.41) | 42.59(39.68-45.49) | 46.76(42.84-50.68) | 19.97(16.05-23.90) | 18.87(14.95-22.79) |
| dLDL, mg/dL |  |  |  |  |  |  |
| < 40 | 10.83(9.00-12.65) | 10.00(8.17-11.82) | 8.05(6.22-9.88) | 27.28(18.76-35.81) | 21.18(12.66-29.70) | 18.30(9.78-26.82) |
| 40 - 70 | 9.14(8.87-9.40) | 8.18(7.91-8.44) | 7.74(7.48-8.01) | 9.65(9.19-10.11) | 7.45(6.99-7.91) | 6.99(6.53-7.45) |
| 70 - 100 | 8.85(8.75-8.95) | 7.68(7.58-7.78) | 7.83(7.74-7.93) | 8.74(8.60-8.89) | 6.64(6.50-6.78) | 6.79(6.65-6.94) |
| 100 - 130 | 10.11(10.04-10.19) | 8.64(8.56-8.71) | 9.00(8.93-9.08) | 9.11(9.02-9.21) | 7.12(7.03-7.22) | 7.34(7.25-7.44) |
| 130 - 160 | 12.27(12.17-12.36) | 10.40(10.30-10.50) | 10.90(10.80-11.00) | 9.91(9.79-10.03) | 8.10(7.98-8.22) | 8.29(8.17-8.41) |
| 160 - 190 | 14.84(14.66-15.01) | 12.76(12.58-12.93) | 13.30(13.12-13.47) | 10.61(10.39-10.83) | 9.26(9.04-9.47) | 9.31(9.09-9.53) |
| 190 - 220 | 17.21(16.78-17.65) | 15.26(14.82-15.69) | 15.85(15.41-16.29) | 11.78(11.19-12.38) | 10.95(10.36-11.54) | 10.84(10.25-11.44) |
| ≥ 220 | 19.25(18.21-20.29) | 17.77(16.73-18.81) | 18.76(17.72-19.79) | 13.27(11.54-14.99) | 13.05(11.32-14.78) | 12.82(11.10-14.55) |

**Supplementary Table 3.** MAD scores between direct LDL-C and different estimated LDL-C values for various TG and LDL-C levels in dyslipidaemia subjects (*for Supplementary Figure 2)*

| MAD, mg/dL (95% CI) | GSHC | | | KOICA | | |
| --- | --- | --- | --- | --- | --- | --- |
|  | Friedewald | Martin | Sampson | Friedewald | Martin | Sampson |
| TG, mg/dL |  |  |  |  |  |  |
| 0 - 100 | 9.10(8.95-9.25) | 9.10(8.95-9.25) | 9.36(9.21-9.51) | 6.87(6.71-7.03) | 7.24(7.09-7.40) | 6.84(6.69-7.00) |
| 100 - 200 | 12.31(12.22-12.41) | 10.59(10.49-10.69) | 10.81(10.72-10.91) | 10.05(9.93-10.17) | 8.01(7.89-8.13) | 8.12(8.00-8.24) |
| 200 - 300 | 19.20(19.00-19.40) | 13.49(13.29-13.70) | 15.42(15.22-15.62) | 15.18(14.96-15.40) | 8.27(8.05-8.49) | 10.55(10.33-10.77) |
| 300 - 400 | 29.23(28.69-29.77) | 17.90(17.36-18.44) | 22.41(21.87-22.94) | 21.17(20.61-21.73) | 9.54(8.98-10.10) | 13.49(12.93-14.05) |
| 400 - 500 | 39.31(38.09-40.52) | 23.05(21.83-24.26) | 29.40(28.19-30.62) | 26.38(25.27-27.49) | 12.58(11.47-13.69) | 15.73(14.63-16.84) |
| 500 - 600 | 49.85(47.63-52.08) | 26.85(24.62-29.08) | 35.82(33.59-38.05) | 32.19(29.82-34.57) | 14.70(12.32-17.08) | 18.27(15.89-20.65) |
| ≥ 600 | 72.82(69.92-75.73) | 35.50(32.60-38.41) | 42.59(39.68-45.49) | 46.76(42.84-50.68) | 19.97(16.05-23.90) | 18.87(14.95-22.79) |
| dLDL, mg/dL |  |  |  |  |  |  |
| < 40 | 16.26(11.91-20.61) | 14.33(9.98-18.68) | 8.24(3.89-12.59) | 29.86(19.81-39.91) | 20.29(10.24-30.34) | 15.43(5.38-25.48) |
| 40 - 70 | 15.10(14.30-15.91) | 12.55(11.75-13.35) | 10.30(9.50-11.11) | 12.45(11.68-13.23) | 8.29(7.52-9.06) | 7.41(6.64-8.18) |
| 70 - 100 | 14.27(13.97-14.57) | 10.55(10.25-10.85) | 10.72(10.42-11.03) | 11.13(10.89-11.38) | 6.93(6.68-7.18) | 7.42(7.17-7.67) |
| 100 - 130 | 15.34(15.14-15.54) | 10.59(10.39-10.79) | 11.93(11.73-12.14) | 11.78(11.61-11.95) | 7.38(7.21-7.55) | 8.44(8.27-8.61) |
| 130 - 160 | 16.50(16.31-16.70) | 11.92(11.72-12.11) | 14.03(13.84-14.23) | 11.98(11.80-12.17) | 8.29(8.11-8.48) | 9.52(9.34-9.71) |
| 160 - 190 | 14.84(14.66-15.01) | 12.76(12.58-12.93) | 13.30(13.12-13.47) | 10.61(10.39-10.83) | 9.26(9.04-9.47) | 9.31(9.09-9.53) |
| 190 - 220 | 17.21(16.78-17.65) | 15.26(14.82-15.69) | 15.85(15.41-16.29) | 11.78(11.19-12.38) | 10.95(10.36-11.54) | 10.84(10.25-11.44) |
| ≥ 220 | 19.25(18.21-20.29) | 17.77(16.73-18.81) | 18.76(17.72-19.79) | 13.27(11.54-14.99) | 13.05(11.32-14.78) | 12.82(11.10-14.55) |

**Supplementary Table 4.** Lipid measurements

|  | **GSHC** | | | **KOICA** | | | | | | | |
| --- | --- | --- | --- | --- | --- | --- | --- | --- | --- | --- | --- |
|  |  |  |  | **Severance Check-up Healthcare Center** | | **Seoul National University Healthcare System Gangnam Center** | | | | **Samsung Medical Center** | |
| **Lipids** | **Date** | **Analyzer** | **Reagent** | Same as GSHC | | **Analyzer** | | | **Reagent** | **Analyzer** | **Reagent** |
| Cholesterol | ~2014.3.17 | Hitachi 7600 | Sekisui |  |  | Architect Ci8200 | | | Abbott | Modular D2400 | Roche |
| Triglyceride | ~2014.3.17 | Hitachi 7600 | Roche |  |  | Architect Ci8200 | | | Abbott | Modular D2400 | Roche |
| LDL-C | ~2014.3.17 | Hitachi 7600 | Sekisui |  |  | Architect Ci8200 | | | Abbott | Modular D2400 | Roche |
| HDL-C | ~2014.3.17 | Hitachi 7600 | Sekisui |  |  | Architect Ci8200 | | | Abbott | Modular D2400 | Roche |
|  | **Date** | **Analyzer** | **Reagent** |  |  | |  |  |  |  |  |
| Cholesterol | 2014.3.18~2014.12.22 | AU5800(Beckman) | Beckman |  |  | |  |  |  |  |  |
| Triglyceride | 2014.3.18~Current | AU5800(Beckman) | Beckman |  |  | |  |  |  |  |  |
| LDL-C | 2014.3.18~Current | AU5800(Beckman) | Beckman |  |  | |  |  |  |  |  |
| HDL-C | 2014.3.18~Current | AU5800(Beckman) | Beckman |  |  | |  |  |  |  |  |
|  | **Date** | **Analyzer** | **Reagent** |  |  | |  |  |  |  |  |
| Cholesterol | 2014.12.23~Current | AU5800(Beckman) | Sekisui |  |  | |  |  |  |  |  |

The inter- and intra-assay coefficients of variation for quality control specimens were <5% for all blood variables.[^1-5^](#_ENREF_1)

**Supplementary References.**

1 Miida, T. *et al.* Homogeneous Assays for LDL-C and HDL-C are Reliable in Both the Postprandial and Fasting State. *J Atheroscler Thromb* **24**, 583-599, doi:10.5551/jat.40006 (2017).

2 Waniek, S. *et al.* Vitamin E (alpha- and gamma-Tocopherol) Levels in the Community: Distribution, Clinical and Biochemical Correlates, and Association with Dietary Patterns. *Nutrients* **10**, doi:10.3390/nu10010003 (2017).

3 Miida, T. *et al.* A multicenter study on the precision and accuracy of homogeneous assays for LDL-cholesterol: comparison with a beta-quantification method using fresh serum obtained from non-diseased and diseased subjects. *Atherosclerosis* **225**, 208-215, doi:10.1016/j.atherosclerosis.2012.08.022 (2012).

4 Yoon, Y. A. *et al.* Standardization Status of Total Cholesterol Concentration Measurement: Analysis of Korean External Quality Assessment Data. *Ann Lab Med* **41**, 366-371, doi:10.3343/alm.2021.41.4.366 (2021).

5 Sung, K. C. *et al.* Comparison of Low-Density Lipoprotein Cholesterol Concentrations by Direct Measurement and by Friedewald Calculation. *Am J Cardiol* **125**, 866-873, doi:10.1016/j.amjcard.2019.12.036 (2020).
