# Supplementary material for: Comparison of the effectiveness of Martin’s equation, Friedewald’s equation, and a Novel equation in low-density lipoprotein cholesterol estimation
Source: Sci Rep. 2021 Jun 29;11:13545. doi: 10.1038/s41598-021-92625-x (PMC8241859; doi:10.1038/s41598-021-92625-x)
Supplement: Supplementary file 2 — Supplementary Information 2. [file 41598_2021_92625_MOESM2_ESM.docx]

**Comparison of the Effectiveness of Martin’s Equation, Friedewald’s Equation, and a Novel Equation in Low-Density Lipoprotein Cholesterol Estimation**

*Running title: Novel and known formulas for LDL-C estimation*

Youhyun Song, M.D.^†^, Hye Sun Lee, Ph.D.^†^, Su Jung Baik, M.D., Soyoung Jeon, M.S., Donghee Han, M.D., Su-Yeon Choi, M.D., Ph.D., Eun Ju Chun, M.D., Ph.D., Hae-Won Han, M.D., Ph.D., Sung Hak Park, M.D., Ph.D., Jidong Sung, M.D., Hae Ok Jung, M.D., Ph.D.,

Ji Won Lee, M.D., Ph.D.^*^, and Hyuk-Jae Chang, M.D., Ph.D.^*^

**Supplemental Material: File 2 *(analysis excluding outliers)***

**Supplementary Figure 4.** Flowchart of study design.

**Supplementary Figure 5.** Scatter plots showing the correlation of direct LDL-C values with estimated LDL-C values using the Friedewald, Martin/Hopkins, and Sampson equations.

**Supplementary Figure 6.** Residual error plots for LDL-C by different equations.

**A)** Severe hyperTG/ TG < 400 mg/dL; **B)** High/Low LDL-C

**Supplementary Figure 7.** Comparison of the mean absolute difference scores between direct LDL-C and different estimated LDL-C values for various TG and LDL-C levels.

**Supplementary Figure 8.** Scatter plots showing the correlation of direct LDL-C values with estimated LDL-C values using the Friedewald, Martin/Hopkins, and Sampson equations in dyslipidaemia subjects.

**Supplementary Figure 9.** Residual error plots for LDL-C by different equations in dyslipidaemia subjects.

**A)** Severe hyperTG/ TG < 400 mg/dL; **B)** High/Low LDL-C

**Supplementary Figure 10.** Comparison of the mean absolute difference scores between direct LDL-C and different estimated LDL-C values for various TG and LDL-C levels in dyslipidaemia subjects.

**Supplementary Table 5.** Distribution of lipid parameters of the study populations.

**Supplementary Table 6.** MAD scores between direct LDL-C and estimated LDL-Cs in severe hyperTG ( ≥ 400 mg/dL) samples.

**Supplementary Table 7.** MAD scores between direct LDL-C and different estimated LDL-C values for various TG and LDL-C levels. (*for Supplementary Figure 7*)

**Supplementary Table 8.** MAD scores between direct LDL-C and different estimated LDL-C values for various TG and LDL-C levels in dyslipidaemia subjects. (*for Supplementary Figure 10)*

**Supplementary Figure 4.** Flowchart of study design.


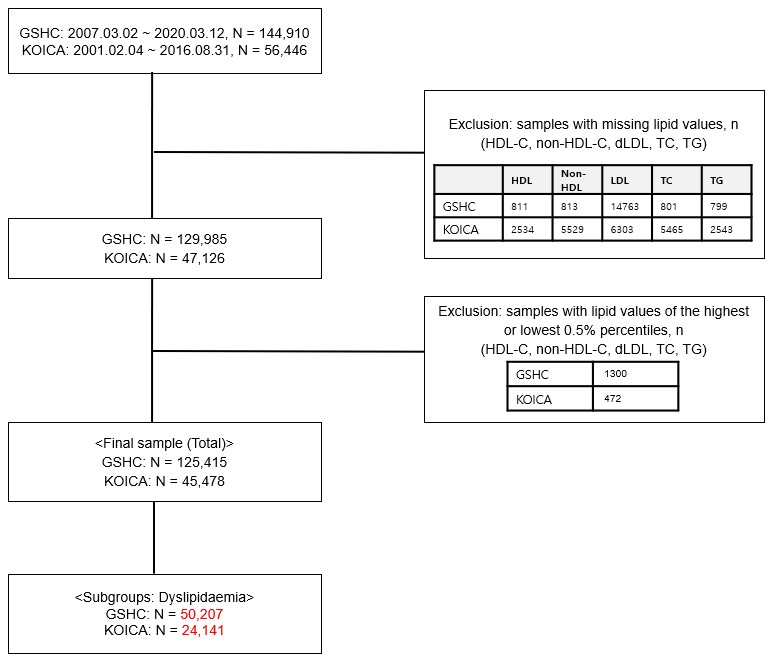


**Supplementary Figure 5.** Scatter plots showing the correlation of direct LDL-C values with estimated LDL-C values using the Friedewald, Martin/Hopkins, and Sampson equations.


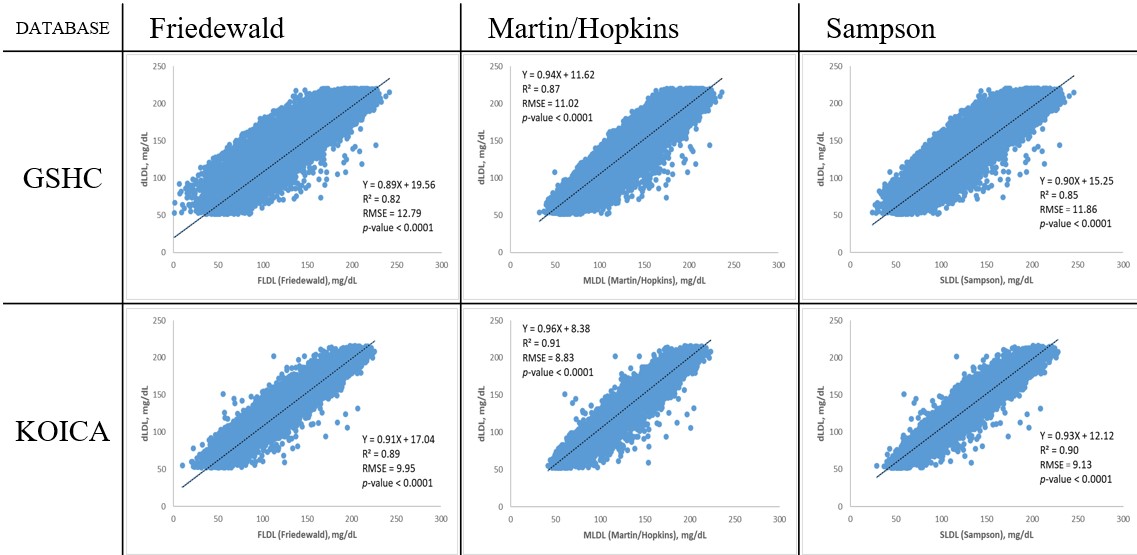


**Supplementary Figure 6.** Residual error plots for LDL-C by different equations.

1. Severe hyperTG/ TG < 400 mg/dL


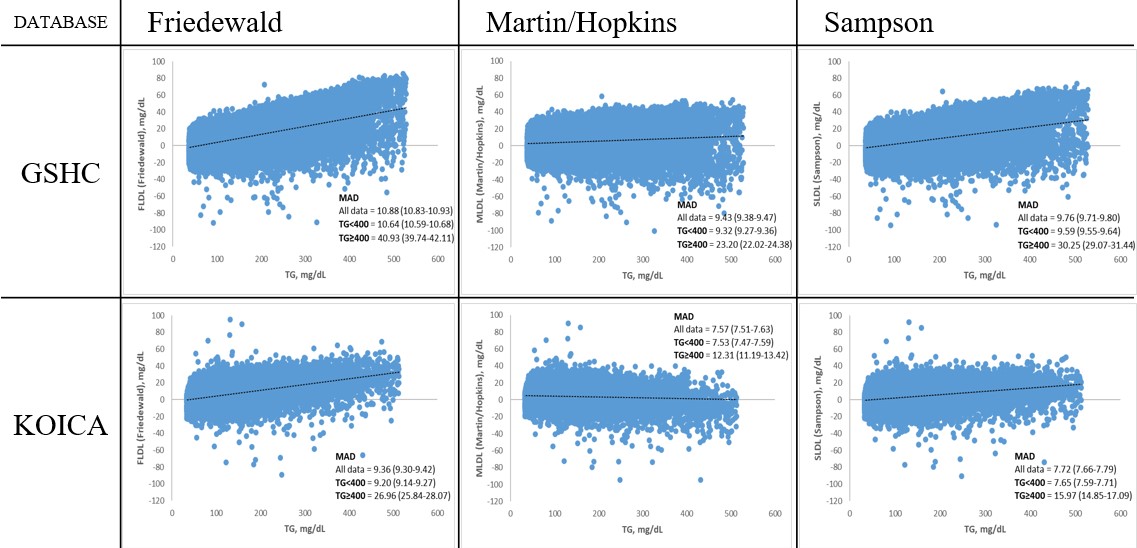


**B)** High/Low LDL-C


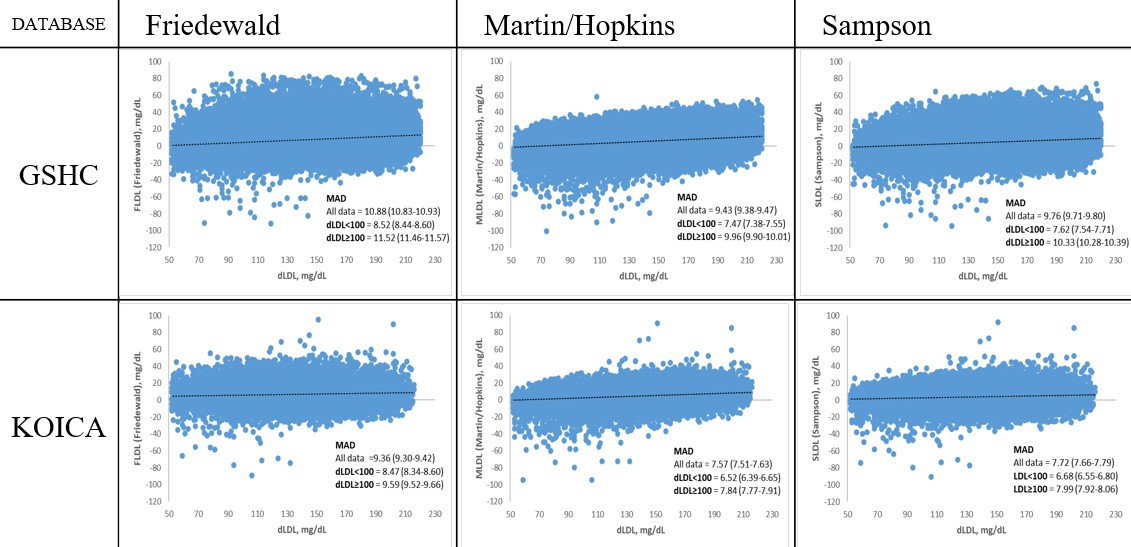


**Supplementary Figure 7.** Comparison of the mean absolute difference scores between direct LDL-C and different estimated LDL-C values for various TG and LDL-C levels.


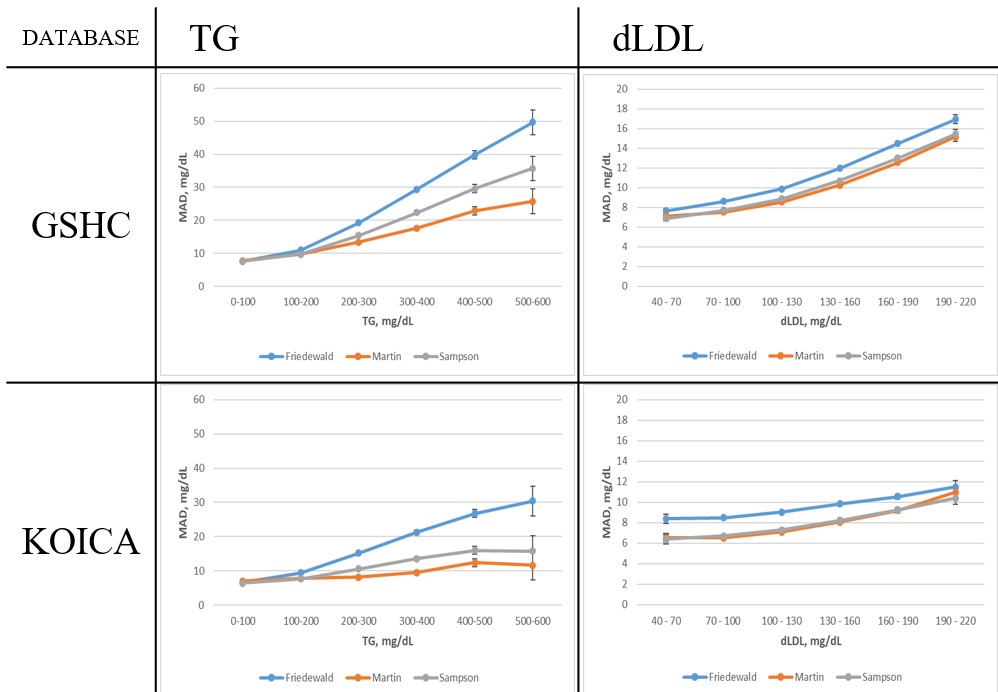


**Supplementary Figure 8.** Scatter plots showing the correlation of direct LDL-C values with estimated LDL-C values using the Friedewald, Martin/Hopkins, and Sampson equations in dyslipidaemia subjects.


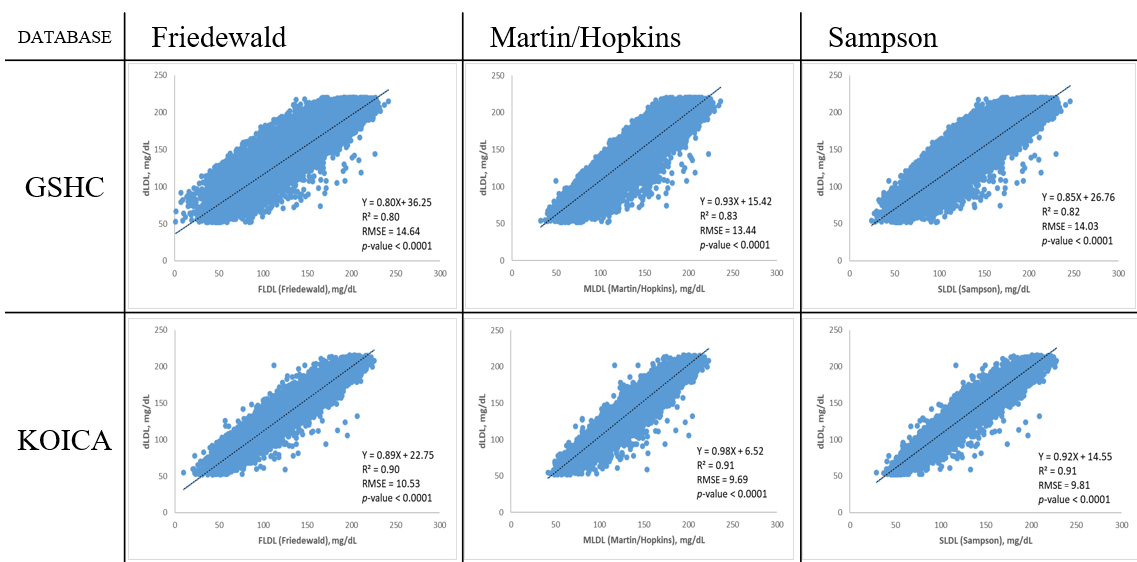


**Supplementary Figure 9.** Residual error plots for LDL-C by different equations in dyslipidaemia subjects.

**A)** Severe hyperTG/ TG < 400 mg/dL


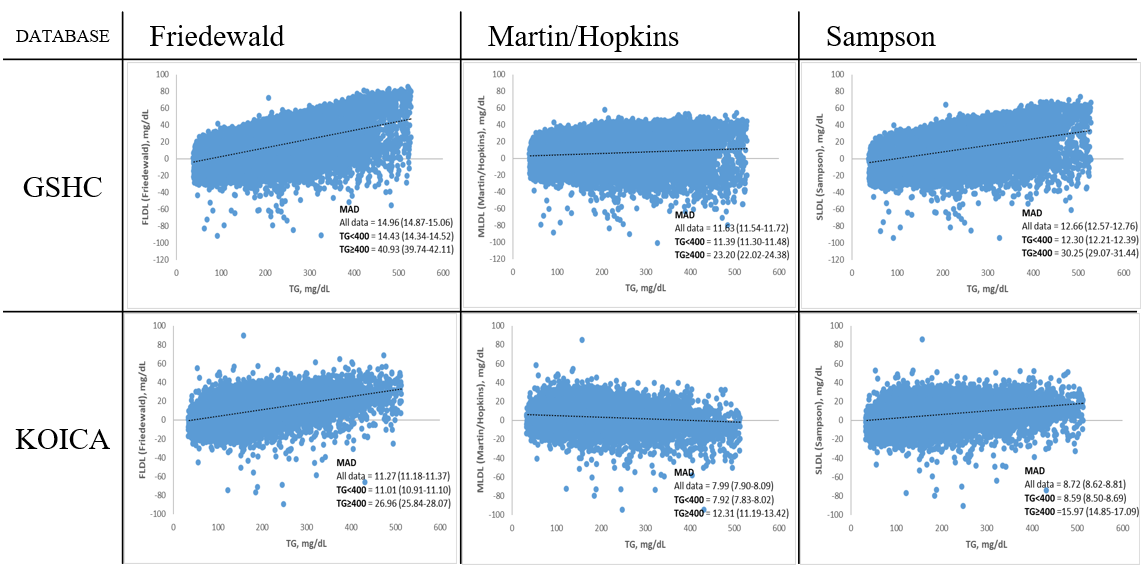


**B)** High/Low LDL-C


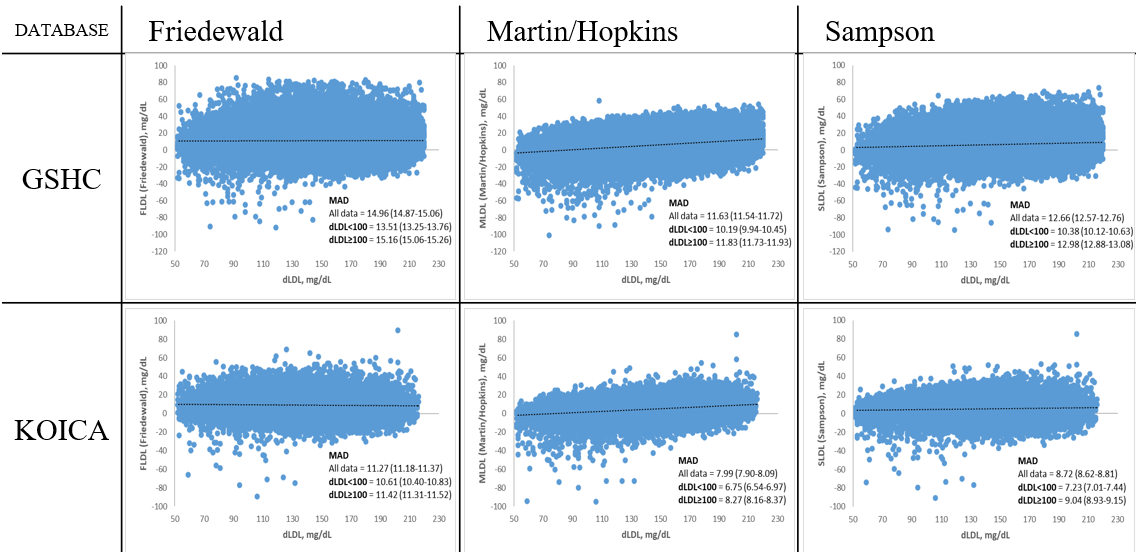


**Supplementary Figure 10.** Comparison of the mean absolute difference scores between direct LDL-C and different estimated LDL-C values for various TG and LDL-C levels in dyslipidaemia subjects.


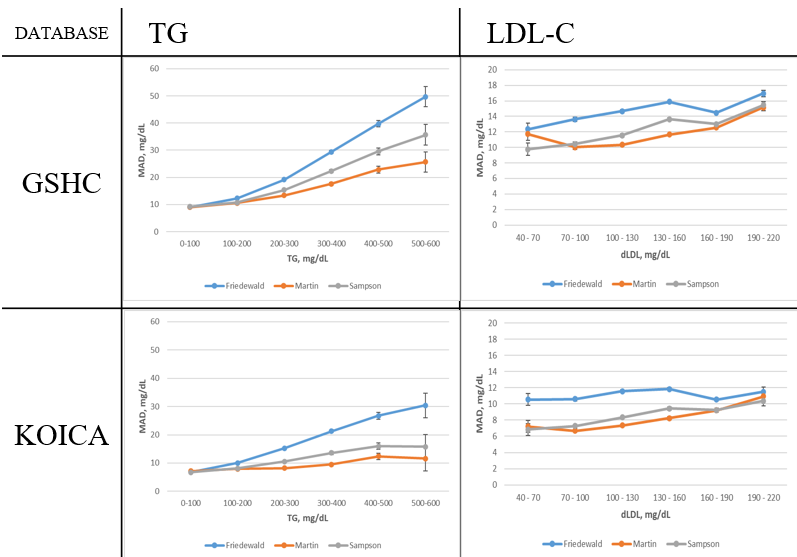


**Supplementary Table 5.** Distribution of lipid parameters of the study populations.

| **Database** | **Total Cohort** | | | **Dyslipidaemia** | | |
| --- | --- | --- | --- | --- | --- | --- |
|  | **Range** | **Mean (SD)** | **Median**  **(25^th^–75^th^ percentile)** | **Range** | **Mean (SD)** | **Median**  **(25^th^–75^th^ percentile)** |
| **GSHC** | | | | | | |
| Cases, n (%) | 125,415 (100.00) | | | 50,207 (40.03) | | |
| Male, n (%) | 66,996 (53.42) | | | 34,037 (67.79) | | |
| Age, year | 12-95 | 48.56 (11.43) | 49 (41-56) | 12-91 | 50.11 (10.39) | 50 (43-57) |
| HDL-C, mg/dL | 29-97 | 54.31 (12.48) | 53 (45-62) | 29-97 | 49.07 (12.32) | 47 (39-56) |
| Non-HDL-C, mg/dL | 65-253 | 143.72 (33.99) | 142 (119-167) | 66-253 | 166.2 (33.2) | 169 (143-189) |
| Direct LDL-C, mg/dL | 52-220 | 125.03 (30.46) | 123 (103-145) | 52-220 | 141.25 (32.9) | 143 (117-166) |
| TC, mg/dL | 114-309 | 198.04 (34.46) | 196 (173-221) | 114-309 | 215.26 (38.24) | 218 (187-244) |
| TG, mg/dL | 37-528 | 124.46 (70.08) | 105 (76-152) | 37-528 | 178.32 (79.29) | 167 (123-215) |
| Cases by TG range, n (%) | | | | | | |
| 0-400 mg/dL | 124411 (99.2) | | | 49203 (98) | | |
| ≥ 400 mg/dL | 1004 (0.8) | | | 1004 (2) | | |
| Cases by LDL-C range, n (%) | | | | | | |
| 40-100 mg/dL | 26716 (21.3) | | | 6058 (12.07) | | |
| ≥ 100 mg/dL | 98699 (78.7) | | | 44149 (87.93) | | |
| Estimated LDL-C Values | | | | | | |
| FLDL, mg/dL | 1-241.6 | 118.83 (31.15) | 117.2 (96.6-139.4) | 1-241.6 | 130.54 (36.63) | 131.8 (103.2-159) |
| MLDL, mg/dL | 32.59-236.45 | 120.57 (30.2) | 118.9 (98.81-140.74) | 32.59-236.45 | 136 (32.45) | 137.67 (111.98-160.36) |
| SLDL, mg/dL | 24.58-245.82 | 121.45 (31.04) | 119.78 (99.11-142.07) | 24.58-245.82 | 134.77 (35.02) | 135.83 (108.59-161.64) |
| **KOICA** | | | | | | |
| Cases, n (%) | 45,478 (100.00) | | | 24,141 (53.08) | | |
| Male, n (%) | 34,677 (76.25) | | | 19,405 (80.38) | | |
| Age, year | 17-97 | 54.07 (8.85) | 53 (48-59) | 17-97 | 54.05 (8.5) | 53 (48-59) |
| HDL-C, mg/dL | 28-97 | 52.2 (12.38) | 50 (43-60) | 28-97 | 48.38 (11.96) | 46 (39-55) |
| Non-HDL-C, mg/dL | 66-248 | 144.68 (32.52) | 143 (121-166) | 66-248 | 156.77 (33.95) | 158 (132-182) |
| Direct LDL-C, mg/dL | 52-216 | 124.92 (29.45) | 124 (104-145) | 52-216 | 132.1 (32.64) | 132 (107-158) |
| TC, mg/dL | 115-301 | 196.88 (32.71) | 196 (174-219) | 115-301 | 205.15 (36.34) | 205 (178-233) |
| TG, mg/dL | 33-513 | 130.81 (72.31) | 113 (80-162) | 33-513 | 167.35 (79.22) | 158 (109-206) |
| Cases by TG range, n (%) | | | | | | |
| 0-400 mg/dL | 45076 (99.12) | | | 23739 (98.33) | | |
| ≥ 400 mg/dL | 402 (0.88) | | | 402 (1.67) | | |
| Cases by LDL-C range, n (%) | | | | | | |
| 40-100 mg/dL | 9286 (20.42) | | | 4324 (17.91) | | |
| ≥ 100 mg/dL | 36192 (79.58) | | | 19817 (82.09) | | |
| Estimated LDL-C Values | | | | | | |
| FLDL, mg/dL | 10-225.6 | 118.52 (30.45) | 117.6 (97-138.8) | 10-225.6 | 123.3 (34.84) | 121.6 (97-150.2) |
| MLDL, mg/dL | 41.74-223.11 | 120.8 (29.12) | 119.65 (100.17-140.17) | 41.74-223.11 | 128.24 (31.83) | 127.41 (104.26-152.47) |
| SLDL, mg/dL | 29.16-228.15 | 121.34 (30.12) | 120.24 (99.92-141.46) | 29.16-228.15 | 127.24 (33.7) | 125.56 (101.79-153.01) |

SD, standard deviation; n, number; HDL-C, high-density lipoprotein cholesterol; Non-HDL-C, non-high-density lipoprotein cholesterol; TC, total cholesterol; LDL-C; low-density lipoprotein cholesterol; dLDL, direct LDL-C; TG, triglyceride; GSHC, Gangnam Severance Hospital Check-up; KOICA, Korea Initiatives on Coronary Artery Calcification; FLDL, LDL-C estimated by Friedewald’s equation; MLDL, LDL-C estimated by Martin/Hopkins equation; SLDL, LDL-C estimated by Sampson’s equation.

Values are presented as mean (standard deviation), median (interquartile range), or as number (%).

**Supplementary Table 6.** MAD scores between direct LDL-C and estimated LDL-Cs in severe hyperTG ( ≥ 400 mg/dL) samples.

| **Mean absolute difference, mg/dL** | | | | | | |
| --- | --- | --- | --- | --- | --- | --- |
| *Database* | GSHC | | | KOICA | | |
| *Equation* | Friedewald | Martin | Sampson | Friedewald | Martin | Sampson |
| *LDL-C strata, mg/dL* | | | | | | |
| 40-70 | 19.838 | 24.846 | 10.735 | 19.779 | 27.75 | 14.162 |
| 70-100 | 24.437 | 22.645 | 15.164 | 25.196 | 14.587 | 12.37 |
| 100-130 | 37.018 | 20.884 | 25.321 | 26.736 | 11.185 | 14.608 |
| 130-160 | 49.272 | 22.224 | 37.071 | 28.964 | 8.503 | 19.579 |
| 160-190 | 52.416 | 25.305 | 43.208 | 31.9 | 9.292 | 25.434 |
| 190-220 | 62.692 | 37.985 | 56.056 | 44.85 | 22.623 | 40.46 |

**Supplementary Table 7.** MAD scores between direct LDL-C and different estimated LDL-C values for various TG and LDL-C levels. (*for Supplementary Figure 7*)

| MAD, mg/dL (95% CI) | GSHC | | | KOICA | | |
| --- | --- | --- | --- | --- | --- | --- |
|  | Friedewald | Martin | Sampson | Friedewald | Martin | Sampson |
| TG, mg/dL |  |  |  |  |  |  |
| 0-100 | 7.61(7.56-7.65) | 7.73(7.68-7.78) | 7.62(7.58-7.67) | 6.58(6.50-6.65) | 6.95(6.88-7.03) | 6.45(6.37-6.52) |
| 100-200 | 10.99(10.92-11.05) | 9.68(9.62-9.75) | 9.74(9.68-9.81) | 9.42(9.33-9.51) | 7.78(7.69-7.87) | 7.69(7.60-7.78) |
| 200-300 | 19.20(19.00-19.41) | 13.35(13.15-13.55) | 15.35(15.15-15.55) | 15.21(14.99-15.43) | 8.18(7.96-8.40) | 10.53(10.31-10.75) |
| 300-400 | 29.39(28.85-29.93) | 17.65(17.11-18.19) | 22.34(21.80-22.88) | 21.26(20.72-21.80) | 9.47(8.93-10.01) | 13.57(13.02-14.11) |
| 400-500 | 39.77(38.54-41.01) | 22.87(21.64-24.11) | 29.54(28.30-30.77) | 26.73(25.58-27.89) | 12.35(11.20-13.51) | 15.98(14.82-17.14) |
| 500-600 | 49.67(45.95-53.40) | 25.71(21.98-29.43) | 35.70(31.97-39.42) | 30.38(26.03-34.72) | 11.61(7.26-15.95) | 15.81(11.47-20.16) |
| dLDL, mg/dL |  |  |  |  |  |  |
| 40 - 70 | 7.63(7.38-7.88) | 7.12(6.88-7.37) | 6.88(6.63-7.13) | 8.38(7.94-8.81) | 6.52(6.09-6.96) | 6.38(5.95-6.82) |
| 70 - 100 | 8.62(8.53-8.71) | 7.51(7.42-7.60) | 7.71(7.62-7.80) | 8.48(8.35-8.61) | 6.52(6.39-6.65) | 6.71(6.58-6.84) |
| 100 - 130 | 9.89(9.82-9.96) | 8.55(8.48-8.62) | 8.87(8.80-8.94) | 9.02(8.92-9.11) | 7.09(7.00-7.19) | 7.30(7.20-7.39) |
| 130 - 160 | 11.96(11.87-12.06) | 10.27(10.18-10.36) | 10.70(10.60-10.79) | 9.83(9.71-9.95) | 8.07(7.96-8.19) | 8.24(8.12-8.35) |
| 160 - 190 | 14.47(14.30-14.64) | 12.56(12.39-12.73) | 13.00(12.84-13.17) | 10.55(10.34-10.77) | 9.20(8.98-9.42) | 9.25(9.04-9.47) |
| 190 - 220 | 16.94(16.50-17.38) | 15.16(14.73-15.60) | 15.46(15.02-15.89) | 11.50(10.89-12.11) | 10.96(10.34-11.57) | 10.40(9.78-11.01) |

**Supplementary Table 8.** MAD scores between direct LDL-C and different estimated LDL-C values for various TG and LDL-C levels in dyslipidaemia subjects. (*for Supplementary Figure 10)*

| MAD, mg/dL (95% CI) | GSHC | | | KOICA | | |
| --- | --- | --- | --- | --- | --- | --- |
|  | Friedewald | Martin | Sampson | Friedewald | Martin | Sampson |
| TG, mg/dL |  |  |  |  |  |  |
| 0-100 | 9.00(8.85-9.16) | 9.03(8.88-9.18) | 9.23(9.08-9.38) | 6.80(6.64-6.95) | 7.17(7.01-7.33) | 6.75(6.59-6.91) |
| 100-200 | 12.28(12.19-12.38) | 10.54(10.44-10.63) | 10.76(10.67-10.86) | 10.03(9.91-10.15) | 7.97(7.85-8.09) | 8.08(7.96-8.20) |
| 200-300 | 19.20(19.00-19.41) | 13.35(13.15-13.55) | 15.35(15.15-15.55) | 15.21(14.99-15.43) | 8.18(7.96-8.40) | 10.53(10.31-10.75) |
| 300-400 | 29.39(28.85-29.93) | 17.65(17.11-18.19) | 22.34(21.80-22.88) | 21.26(20.72-21.80) | 9.47(8.93-10.01) | 13.57(13.02-14.11) |
| 400-500 | 39.77(38.54-41.01) | 22.87(21.64-24.11) | 29.54(28.30-30.77) | 26.73(25.58-27.89) | 12.35(11.20-13.51) | 15.98(14.82-17.14) |
| 500-600 | 49.67(45.95-53.40) | 25.71(21.98-29.43) | 35.70(31.97-39.42) | 30.38(26.03-34.72) | 11.61(7.26-15.95) | 15.81(11.47-20.16) |
| dLDL, mg/dL |  |  |  |  |  |  |
| 40 - 70 | 12.36(11.56-13.16) | 11.72(10.92-12.52) | 9.74(8.94-10.54) | 10.55(9.82-11.28) | 7.22(6.50-7.95) | 6.84(6.11-7.56) |
| 70 - 100 | 13.63(13.36-13.90) | 10.03(9.76-10.29) | 10.45(10.18-10.71) | 10.62(10.40-10.84) | 6.69(6.47-6.92) | 7.28(7.05-7.50) |
| 100 - 130 | 14.66(14.48-14.84) | 10.33(10.15-10.51) | 11.55(11.36-11.73) | 11.59(11.42-11.76) | 7.34(7.17-7.50) | 8.36(8.19-8.53) |
| 130 - 160 | 15.88(15.71-16.06) | 11.66(11.48-11.84) | 13.63(13.45-13.81) | 11.85(11.67-12.03) | 8.25(8.06-8.43) | 9.44(9.25-9.62) |
| 160 - 190 | 14.47(14.30-14.64) | 12.56(12.39-12.73) | 13.00(12.84-13.17) | 10.55(10.34-10.77) | 9.20(8.98-9.42) | 9.25(9.04-9.47) |
| 190 - 220 | 16.94(16.50-17.38) | 15.16(14.73-15.60) | 15.46(15.02-15.89) | 11.50(10.89-12.11) | 10.96(10.34-11.57) | 10.40(9.78-11.01) |
